# Resequencing of sweetpotato germplasm resources reveals key loci associated with multiple agronomic traits

**DOI:** 10.1093/hr/uhac234

**Published:** 2022-10-19

**Authors:** Shizhuo Xiao, Xibin Dai, Lingxiao Zhao, Zhilin Zhou, Lukuan Zhao, Pan Xu, Bingqian Gao, An Zhang, Donglan Zhao, Rui Yuan, Yao Wang, Jie Wang, Qinglian Li, Qinghe Cao

**Affiliations:** Jiangsu Xuzhou Sweetpotato Research Center/Sweetpotato Research Institute, Chinese Agricultural Academy of Sciences, Xuzhou 221131, China; Jiangsu Xuzhou Sweetpotato Research Center/Sweetpotato Research Institute, Chinese Agricultural Academy of Sciences, Xuzhou 221131, China; Jiangsu Xuzhou Sweetpotato Research Center/Sweetpotato Research Institute, Chinese Agricultural Academy of Sciences, Xuzhou 221131, China; Jiangsu Xuzhou Sweetpotato Research Center/Sweetpotato Research Institute, Chinese Agricultural Academy of Sciences, Xuzhou 221131, China; Jiangsu Xuzhou Sweetpotato Research Center/Sweetpotato Research Institute, Chinese Agricultural Academy of Sciences, Xuzhou 221131, China; College of Pastoral Agriculture Science and Technology, Lanzhou University, Lanzhou 730020, China; Jiangsu Xuzhou Sweetpotato Research Center/Sweetpotato Research Institute, Chinese Agricultural Academy of Sciences, Xuzhou 221131, China; Jiangsu Xuzhou Sweetpotato Research Center/Sweetpotato Research Institute, Chinese Agricultural Academy of Sciences, Xuzhou 221131, China; Jiangsu Xuzhou Sweetpotato Research Center/Sweetpotato Research Institute, Chinese Agricultural Academy of Sciences, Xuzhou 221131, China; Jiangsu Xuzhou Sweetpotato Research Center/Sweetpotato Research Institute, Chinese Agricultural Academy of Sciences, Xuzhou 221131, China; Jiangsu Xuzhou Sweetpotato Research Center/Sweetpotato Research Institute, Chinese Agricultural Academy of Sciences, Xuzhou 221131, China; Jiangsu Xuzhou Sweetpotato Research Center/Sweetpotato Research Institute, Chinese Agricultural Academy of Sciences, Xuzhou 221131, China; Jiangsu Xuzhou Sweetpotato Research Center/Sweetpotato Research Institute, Chinese Agricultural Academy of Sciences, Xuzhou 221131, China; Jiangsu Xuzhou Sweetpotato Research Center/Sweetpotato Research Institute, Chinese Agricultural Academy of Sciences, Xuzhou 221131, China

## Abstract

Sweetpotato is an important crop that exhibits hexaploidy and high heterozygosity, which limits gene mining for important agronomic traits. Here, 314 sweetpotato germplasm resources were deeply resequenced, and 4 599 509 SNPs and 846 654 InDels were generated, among which 196 124 SNPs were nonsynonymous and 9690 InDels were frameshifted. Based on the Indels, genome-wide marker primers were designed, and 3219 of 40 366 primer pairs were selected to construct the core InDel marker set. The molecular ID of 104 sweetpotato samples verified the availability of these primers. The sweetpotato population structures were then assessed through multiple approaches using SNPs, and diverse approaches demonstrated that population stratification was not obvious for most Chinese germplasm resources. As many as 20 important agronomic traits were evaluated, and a genome-wide association study was conducted on these traits. A total of 19 high-confidence loci were detected in both models. These loci included several candidate genes, such as *IbMYB1*, *IbZEP1,* and *IbYABBY1*, which might be involved in anthocyanin metabolism, carotenoid metabolism, and leaf morphogenesis, respectively. Among them, *IbZEP1* and *IbYABBY1* were first reported in sweetpotato. The variants in the promoter and the expression levels of *IbZEP1* were significantly correlated with flesh color (orange or not orange) in sweetpotato. The expression levels of *IbYABBY1* were also correlated with leaf shape. These results will assist in genetic and breeding studies in sweetpotato.

## Introduction

Sweetpotato (*Ipomoea batatas* [L.] Lam.) is an important food, feed, and energy crop that is widely grown in more than 100 countries and regions around the world. China produces the most sweetpotatoes every year, accounting for more than half of the world’s total yield [1]. In addition to the high production of carbohydrates, the tuberous roots of sweetpotato are also rich in multiple nutrients, including protein, fiber, and vitamins, especially anthocyanins and carotenoids [2], whose roles in health care have drawn increasing attention in recent years [3].

Sweetpotato belongs to the *Ipomoea* genus of Convolvulaceae and is widely believed to have originated in tropical America, likely Peru or Mexico [4]. Sweetpotato is hexaploid (2n = 6x = 90), and it is generally believed to be autohexaploid, although this question has not been clearly explored [5,6]. Several wild species may be involved in the origin and evolution of sweetpotato, among which the diploid *Ipomoea trifida* is thought to be one of its ancestors [7,8]. Due to the complexity of the genome, the *de novo* assembly of the sweetpotato genome is lacking. In 2018, high-quality genome maps of two diploid wild relatives of sweetpotato, *I. trifida* and *Ipomoea triloba*, were released, and the genome of *I. trifida* has been widely used as a reference sequence in whole genome studies [7]. In addition, some other genomes, such as the haplotype-resolved sweetpotato genome by new algorithms [8] and the genome of wild species *I. nil* [9], have been sequenced and assembled. However, these are not suitable reference genomes for whole genome studies in sweetpotato. In 2014, the organization jointly established by China, Japan, and South Korea launched the *de novo* assembly of sweetpotato cultivar “Xushu18” [ 10–12]. At present, a high-quality assembly at the chromosome level has been obtained, and six sets of subgenomes with 90 chromosomes have been resolved (upcoming released), which can be used as references for genome-wide studies in sweetpotato.

Normally, sweetpotato is vegetatively propagated, and self-incompatibility and hybridization incompatibility are quite widespread, which restricts the construction of a genetic population of sweetpotato. In addition, its hexaploid nature makes molecular markers less specific and readable. A pair of SSR amplification primers can produce up to 46 bands [13]. Due to the absence of a high-quality reference genome, the anchor positions of SSR markers on chromosomes are also indistinct. This explains why gene mapping in sweetpotato has lagged behind diploid crops, such as rice and maize, as well as some polyploid crops, such as wheat, cotton, and potato.

Genome-wide association study (GWAS) is a method for mapping genes using natural populations based on linkage disequilibrium (LD). GWAS does not require genetic populations to be constructed, and it also has advantages, such as higher mapping accuracy, less time-consuming, and more variant-detecting. It has been widely used in human, animal, and plant studies [14–16]. The release of a high-quality reference genome provides new opportunities for GWAS in sweetpotato.

In this study, next-generation sequencing was applied to 314 sweetpotato germplasm resources originating from different countries. The genome-wide variants were detected using the genome of “Xushu18” as a reference. High-throughput and user-friendly InDel markers were designed according to genome-wide variants. The population structure and kinship of these individuals were elucidated. Finally, we conducted GWAS on 20 key agronomic traits, and several significant loci, including novel loci, were detected. Our study is useful for the genetic study and molecular breeding of sweetpotato.

## Results

### Resequencing of 314 sweetpotato germplasm resources and variant discovery

A total of 314 sweetpotato germplasm resources originating from different countries were obtained, including accessions, landraces, and breeding lines, and most of them were from China (Table S1). The whole genomes of 314 samples were resequenced using the Nova-Seq 6000 platform, generating 6081.67 Gb of raw data in total, with an average of 19.37 Gb for each sample and 6048.13 Gb of clean data after filtration, with an average of 19.26 Gb for each sample (Table S1). The average sequencing depth reached 41.69-fold, referencing the diploid genome [7].

**Figure 1:**
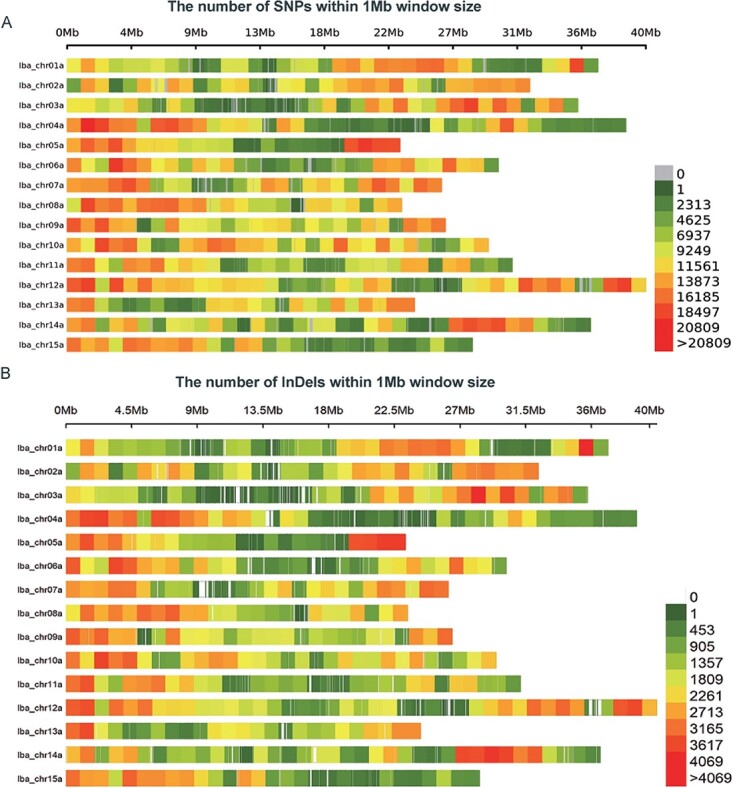
**Genome-wide variant calling. A** Distribution of SNPs on 15 chromosomes. **B** Distribution of InDels on 15 chromosomes. “Iba_chr” means chromosome.

Considering the high similarity among the six sets of subgenomes of sweetpotato, one set of subgenomes (Iba_chr01a–Iba_chr15a) from “Xushu18” was selected as the reference, and the reads were aligned to the reference to mine the genome-wide variants. After quality control and screening, 4 599 509 SNPs and 846 654 InDels were obtained (Fig. 1A and 1B). Most variants (2 441 858 SNPs and 460 634 InDels) were located in intergenic regions, and the second most variants (839 747 SNP and 180 700 InDels) were located in introns. There were 557 793 SNPs and 20 676 InDels located in the exons, among which about one-third of SNPs were nonsynonymous, and about half of InDels were frameshifted (Table S2). Owing to the autopolyploidy of sweetpotato and the subgenome for reference, the impacts of variants, especially in the coding region, may be inaccurately estimated, but the results are still instructive.

### Construction of a core InDel marker set in sweetpotato

To produce user-friendly molecular markers, we screened the InDels according to the following rules: i) only bi-allelic InDels were kept; ii) only InDels whose base differences were not less than five were kept; and iii) InDels with a heterozygosity of more than 80% or minor allele frequency (MAF) of less than 0.2 were filtered out to avoid less polymorphisms among most samples. Finally, 47 961 InDels were kept for molecular marker design.

**Figure 2:**
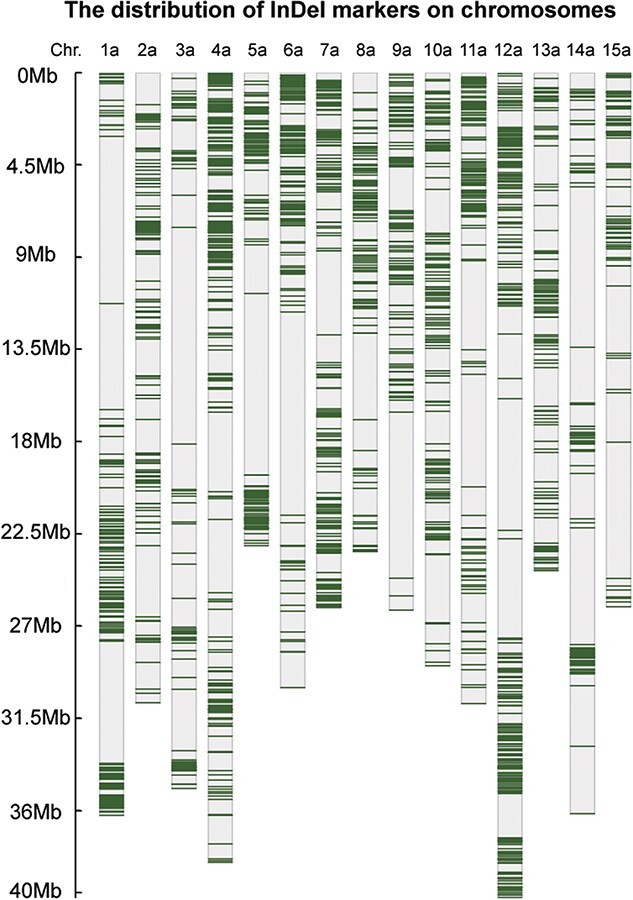
Distribution of InDel markers on chromosomes.

We selected the upstream and downstream 200 bp of InDels as templates to design PCR primers, set the parameters (see the “Materials and methods” section for details), and produced 40 366 primer pairs (Table S3). The sweetpotato genome is rich in repetitive sequences. To guarantee the specificity of the primers, we aligned all primers to the six sets of reference genomes, and only those matched at the targets or allelic loci were retained. More than 92% of the primers were filtered out, and eventually only 3219 primer pairs remained. They were considered excellent core InDel marker sets in sweetpotato (Table S4). On average, more than 214 markers were present on each chromosome, with the average physical distance between adjacent markers being about 140 kb (Fig. 2).

To verify the designed primers, 30 primer pairs from the core set and eight sweetpotato samples as templates were randomly selected for PCR and electrophoresis. When counting the amplified bands, only the target bands were recorded, eliminating the effect of non-specific amplification. Among them, about half of the primers produced relatively simple and polymorphic bands compared to previous SSR primers in sweetpotato [17] (Fig. S1). To further verify the application value of these markers, 104 sweetpotato samples were randomly selected, and 15 primer pairs were selected from the marker set to construct a molecular ID for each sample. The samples were completely distinguished by the markers (Table S5). The construction of a high-quality core InDel marker set provides a powerful tool for genetic and breeding studies in sweetpotato.

### Phylogenetic analysis and population structure of sweetpotato

**Figure 3:**
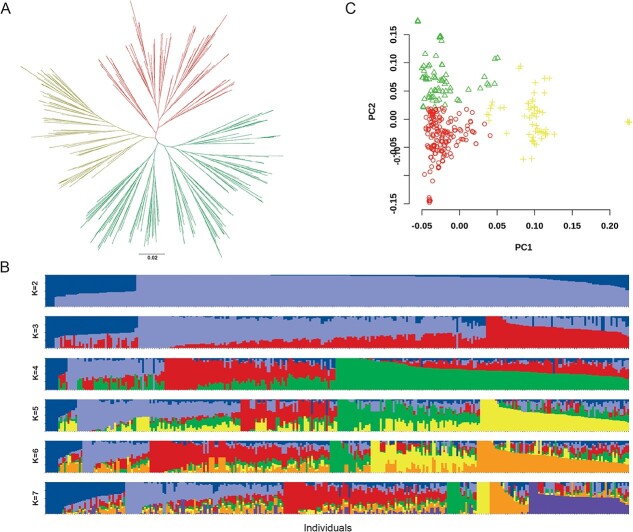
**Population analysis of sweetpotato. A** Phylogenetic tree of sweetpotato. Different colors mean different groups. **B** Population structure analysis. The *k* value ranged from 2 to 7. **C** Principal component analysis (PCA). Different colors mean different groups.

Using the 4 599 509 SNPs, a phylogenetic tree was constructed using the maximum likelihood (ML) method (Fig. 3A and Fig. S2). According to the tree, these samples were divided into three groups, and we studied the relationship between the groups and the origin regions or flesh color of the tuberous roots. Six of nine African introductory germplasm resources were grouped into one clade, four adjacent germplasm resources were from southeast Asia or the Pacific Island, and five of the eight Japanese germplasm resources were also closely related (Fig. S2). Although there were correlations between groups and regions, they were still not strong enough. Such a case was more obvious in Chinese germplasm resources, whose grouping did not significantly correlate with the provinces but rather with the breeding institutions (Fig. S2). In addition, we identified several unsourced landraces based on the trees, such as that “Zhanjiangcheng” may be a derivative of “Pushu32”, and “Wuguzai” may be a same variety with “Nuomishu” (Fig. S2).

In addition, the population structure analysis by stacking diagram showed significant interpenetration among the different populations (Fig. 3B). Principal component analysis (PCA) also demonstrated that the boundaries among the different populations were not clearly distinguished (Fig. 3C). The ambiguous population structure may be a result of the widespread introduction and cross-breeding between different regions, leading to frequent gene exchange of sweetpotato in China.

To further understand the domestication process of sweetpotato, fixation indices (*F_ST_*) were estimated between different groups. No significant differentiation was observed between modern varieties (including accessions and breeding lines) and landraces, but some loci were obviously selected (Fig. S3A). These loci may contain genes related to modern requirements for sweetpotato, such as appearance and health value. *F_ST_* between purple fleshed and no-purple fleshed groups showed that some selected loci coincided with the divergence loci of modern varieties and landraces (Fig. S3B), indicating the genes associated with purple flesh were retained during the breeding process of sweetpotato.

### GWAS on key traits and the discovery of significant loci

A total of 20 valuable agronomic traits were evaluated, which included 12 underground traits: total anthocyanin content (AN), total carotenoid content (CA), β-carotene content (BC), flesh color (purple or not) (FP), flesh color (orange or white) (FO), skin color (SC), dry matter content (DM), crude protein content (CP), starch content (ST), reducing sugar content (RS), soluble sugar content (SS), and weight of tuberous roots per plant (WEI); and eight aboveground traits: apical leaf color (ALC), vine tip pubescence (VTP), leaf shape (LS), number of leaf lobes (LLN), leaf vein color (LVC), vein base color (VBC), petiole base color (PBC), and number of base branches (BBN) (Table S6).

**Figure 4:**
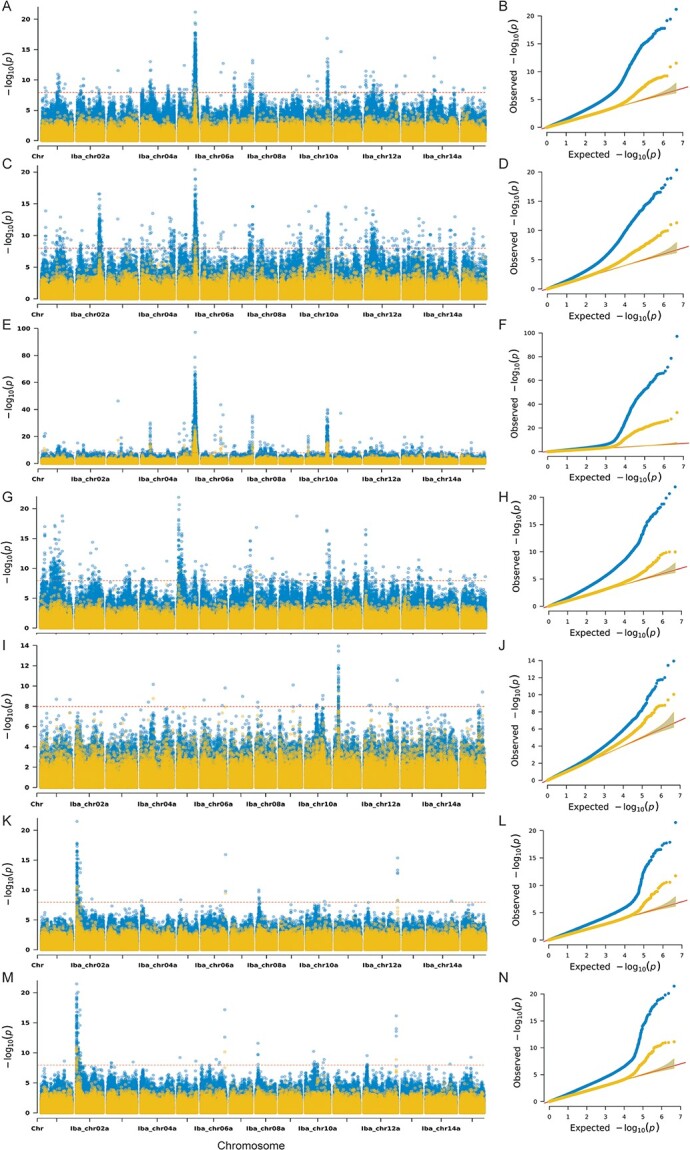
**Significant loci detected using genome-wide association study (GWAS) using both models. A–B** The Manhattan and Quantile-Quantile (Q-Q) plots for SC. **C–D** The Manhattan and Q-Q plots for AN. **E–F** The Manhattan and Q-Q plots for FP. **G–H** The Manhattan and Q-Q plots for ALC. **I–J** The Manhattan and Q-Q plots for FO. **K–L** The Manhattan and Q-Q plots for LS. **M–N** The Manhattan and Q-Q plots for LLN. Blue dots represent general linear model (GLM) and yellow dots represent mixed linear model (MLM), while red dotted lines indicate the threshold value.

We conducted GWAS on these traits using a genome-wide SNP panel using a general linear model (GLM) and mixed linear model (MLM) [18]. The results demonstrated that many SNPs were associated with the traits, and more loci were detected using GLM than MLM. GLM model can obtain more loci, but the false positives may be higher. The MLM model may miss some potential loci, but it can ensure that the detected loci are more reliable. Finally, 19 loci were considered to have high confidence because they were detected using both models and displayed obvious peaks. The associated traits were FP, FO, SC, AN, ALC, LS, and LLN (Table 1, Fig. 4 and Fig. S4–S28).

**Table 1:** Genome-wide significant association signals of agronomic traits. in both models.

Traits	Chromosomes	Position of peak SNP^a^ (bp)	P-value of peak SNP	Ref^b^	Alt^c^	Candidate
						genes
FO	Iba_chr11a	5 208 803	9.06E-11	T	A	*IbZEP1*
AN	Iba_chr02a	27 590 801	2.50E-09	G	A	
	Iba_chr05a	20 431 475	4.89E-12	C	A	*IbMYB1*
	Iba_chr06a	3 680 340	5.55E-09	C	T	
	Iba_chr07a	25 908 048	7.80E-09	G	T	
	Iba_chr10a	25 648 613	6.97E-09	G	A	
FP	Iba_chr04a	10 334 101	7.59E-16	G	A	
	Iba_chr05a	20 357 180	9.72E-34	C	T	*IbMYB1*
	Iba_chr06a	22 924 714	9.13E-20	T	C	
	Iba_chr07a	25 907 848	1.75E-14	G	A	
	Iba_chr10a	25 658 043	7.69E-22	G	T	
	Iba_chr10a	2 664 267	1.66E-13	C	T	
SC	Iba_chr05a	20 276 716	2.96E-12	G	A	*IbMYB1*
ALC	Iba_chr05a	1 500 007	1.07E-10	G	A	
	Iba_chr07a	23 780 071	1.42E-09	C	T	
LS	Iba_chr02a	597 913	1.84E-12	G	A	
	Iba_chr12a	38 790 815	4.47E-09	G	T	*IbYABBY1*
LLN	Iba_chr02a	620 613	7.61E-12	C	T	
	Iba_chr12a	38 790 815	1.32E-09	G	T	*IbYABBY1*

a The peak positions detected by MLM.

b The alleles on reference genome.

c The alterable alleles.

The locus with the highest peak on Iba_chr05a was associated with three anthocyanin-related traits: FP, SC, and AN (Fig. 4A–F). A gene related to anthocyanin content in sweetpotato tuberous roots was indicated at this locus. We attempted to identify the candidate genes responsible for these phenotypes, but no suitablegenes for candidate were found. The R2R3-MYB transcription factor is the dominant factor regulating anthocyanin accumulation in plants [19], as well as in sweetpotato [20–23]. We used BLASTN to align the reported MYB sequence to the reference genome. The flanking sequences of *IbMYB1,* also called *IbMYB1-2null* [20] (NCBI accession AB588639) (Table S7), were located in the mappingregion, which was only about 10 kb from the second highest peak SNP. No coding sequence of *IbMYB1* was identified at this location in reference genome, instead an unannotated sequence (Table S8); however, according to previous reports, the variationin the flanking sequence of *IbMYB1* is responsible for altering anthocyanin content [20]. To some extent, this locus could be considered *IbMYB1* or, at least, *IbMYB1*-related. To verify whether it was a true replacement of coding sequence in reference genome, the boundary sequences of the insertion sequence were amplified by primers (Fig. S29A). The expected PCR products suggest that a replacement existed (Fig. S29B). Several reads by next-generation sequencing covered the boundaries of the inserted sequence, which supported our speculation (Fig. S29C).

We also scanned other loci and found a gene encoding zeaxanthin epoxidase (ZEP) located in the locus on Iba_chr11a associated with FO (Fig. 4I and J). This gene is involved in carotenoid metabolism by catalyzing zeaxanthin converting to violaxanthin in plants [24,25], and we named it *IbZEP1.* The locus associated with LS and LLN on Iba_chr12a contained a *YABBY* gene, which was considered to play an important role in leaf morphogenesis [26,27], and we named it *IbYABBY1* (Fig. 4K–N). Additionally, some loci with high confidence were detected using both models and multiple phenotypes, such as the locus at the end of Iba_chr10a (Fig. 4A, C, E and Fig. S4, S7 and S9) and the locus at the end of Iba_chr7a (Fig. 4C and E and Fig. S4, S7), and we were searching for the candidate genes in these loci.

### Candidate gene analysis

To further study these candidate genes, the distribution of the variants was analyzed. Compared with the gene sequences of reference genome (Table S8), there were 70 variants (58 SNPs and 12 InDels) on the 5′ flanking region, and 10 variants (9 SNPs and 1 InDel) on the 3′ flanking region of *IbMYB1*. *IbZEP1* harbored 131 variants in total, 12 of which were non-synonymous. *IbYABBY1* contained 162 variants, and only two were non-synonymous (Table S9). Further evaluation was performed using PROVEAN [28], and some of the variants showed deleterious effects on the protein (Table S9). To verify the reliability of variants on genes, two samples were selected, and their candidate genes were sequenced using the Sanger method (Fig. S30). It demonstrated that the results of short-read sequencing, alignment, and variant calling were credible.

**Figure 5:**
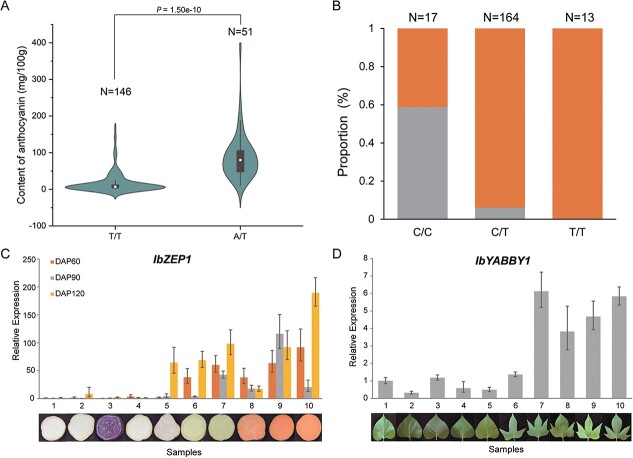
**Haplotypes and expression analysis of candidate genes*.* A** Association between a SNP (19 964 993 bp on Iba_chr5a) and AN. The significance of difference was evaluated by *t*-test. **B** Association between a SNP (5 235 151 bp on Iba_chr11a) and FO. The orange color represents the percentage of orange-fleshed (including yellow-fleshed) sweetpotatoes, and the gray color represents the percentage of white-fleshed sweetpotatoes. N indicates the number. **C** Relative expression of *IbZEP1* in different colored sweetpotatoes at three growth stages. DAP refers to the days after planting. **D** Relative expression of *IbYABBY1* in different shaped leaves of sweetpotato. The data shown are the mean ± SE. The data were normalized to Sample 1, and the samples were described in the Materials and Methods.

The association between these variants and the phenotype was analyzed, and the 19 964 993 bp SNP on Iba_chr5a was significantly correlated with AN. There were two forms at this site: homozygous T (T/T) or heterozygous (A/T). A sample harboring A on this site would accumulate more anthocyanin than that harboring only T, and this meant that the haplotype with A on this site was a high-anthocyanin-content haplotype (Fig. 5A, Fig. S31A). In addition, similar results were observed using FP (Fig. S32).

*IbZEP1* was analyzed in the same way, and the SNP at 5235151 bp on Iba_chr11a was highly correlated with FO. When a sample harbored only T (T/T) at this site, its flesh color was orange (13/13). If the sample simultaneously harbored C and T (C/T) at this site, it had a high probability of being orange-fleshed (154/164). Correspondingly, if a sample harbored only C (C/C) at this site, it had more than half the probability of being white-fleshed (10/17) (Fig. 5B and Fig. S31B). At different stages of root development, the *IbZEP1* expression level was significantly higher in orange-fleshed (including yellow-fleshed) samples than in white- and purple-fleshed samples, and the difference was generally more than 50-fold (Fig. 5C). Considering that the variant at 5235151 bp was located in the promoter region and was differentially expressed, we evaluated the potential effects of this variant on transcription. The results showed that the mutation may affect the binding of some transcription factors, including bZIP, NAC, and MADF (Fig. S33).

The association between variants in *IbYABBY1* and leaf shapes was not clear; however, the expression analysis in leaf shape demonstrated that the expression of *IbYABBY1* was higher in most lobed leaves than in cordate leaves (Fig. 5D). Although there were inconsistencies, such as Sample 6, with comparatively low gene expression and lobed leaves, it was also understandable because this locus was not the only one controlling leaf shape, and the locus on Iba_chr02a was more remarkable (Fig. 4K and M). We were searching for the candidate gene in the loci on Iba_chr02a.

We analyzed the relationship between the candidate genes in sweetpotato and their homologous genes in other plants. In addition to the genus *Ipomoea*, R2R3-MYB genes from Solanaceae are closer to *IbMYB1*, and some species on the evolutionary tree possess purple organs, such as the corolla of *Brunfelsia australis* and *Petunia integrifolia*, and the fruit of *Lycium ruthenicum* (Fig. S34A). *ZEPs* from *Solanum tuberosum* and *Daucus carota* were more closely related to *IbZEP1* (Fig. S34B), suggesting that the *ZEP* of plants with underground tubers or tuberous roots may differ from that of other plants. Plants generally possess multiple *YABBY* family genes [29], and the *IbYABBY1* of sweetpotato was divided into the *YABBY2* family (Fig. S34C). The *YABBY2* genes from other plants were obtained from the database, and the classification of the *YABBY2* gene was more distinct between dicot and monocot plants, whose leaf morphology showed obvious differences, indicating the role of this gene in leaf development (Fig. S34D).

## Discussion

GWAS is an efficient method for detecting important genes, particularly for multiple traits. With the rapid improvement of sequencing technology and algorithms, GWAS has become more and more popular [30, 31]. Sweetpotato is an important food, feed, and energy crop, and it is worthy of understanding the genetic basis of important agronomic traits. Sweetpotato usually reproduces asexually, and the construction of a genetic population of sweetpotato is not ideal due to widespread cross-incompatibility and self-incompatibility, so it is better to use GWAS in sweetpotato. However, as a homologous hexaploid crop, sweetpotato lacked high-quality genomic sequences until now. The genome of diploid wild relative *I. trifida* was used as a reference in previous studies. However, tuberous root-related genes are absent in *I. trifida*, limiting its application in gene mining. In our study, the genome of cultivar “Xushu18” was used as a reference, which could be used to map tuberous-root-related genes. Given the high homology among different subgenomes of auto-hexaploids and the current algorithms for sequence alignment, using the six sets of genomes as a reference filtered out most reads as non-unique matches. Therefore, one set of subgenomes (Iba_chr01a–Iba_chr15a) with the highest quality was selected as the reference. In total, 4 599 509 SNPs and 846 654 InDels were obtained (Fig. 1B and C). The closely related germplasm resources clustered together in phylogenetic analysis, and the reported associated loci verified the credibility of variant calling using GWAS (Fig. S2 and Fig. 4).

Molecular markers based on PCR are the basis of applicationsincluding evolutionary analysis, the construction of molecular IDs, and marker-assisted selection (MAS). Due to the abundantrepetitive sequences in the genome and the lack of a high-quality reference genome for sweetpotato, the previous molecular markers were low-throughput, less readable, non-anchored, and had poor specificity. In addition, previous markers were dominant,ignoring the interaction of alleles [17, 32]. These problems have hindered the development of genetic studies in sweetpotato. In this study, 40 366 pairs of genome-wide molecular markers were designed according to the InDels (Table S3), and a core marker set consisting of 3219 pairs of high-quality primers was constructed after specific screening (Fig. 2 and Table S4). Gel electrophoresisanalysis of randomly selected primers showed that about half of the target bands were clear and polymorphic. The heterozygous or homozygous loci were distinguished, which means they were co-dominant markers (Fig. S1). Molecular IDs for 104 sweetpotatosamples also indicated the practicability of the markers (Table S5). The core marker set will provide strong support for the genetic and breeding study of sweetpotato.

China produces the most sweetpotatoes every year [1]. It is meaningful to clarify the population structure of sweetpotato in China. Although some studies have been done using various markers, but limited by the number of markers, no convincing conclusions were drawn [33,34]. In our study, high-throughput SNPs were used to analyze the kinship and population structure of sweetpotato using multiple methods (Fig. 3). We found that, although the germplasm resources introduced from abroad had a close genetic relationship, they were not completely differentiated from the domestic germplasm resources (Fig. 3A and Fig. S2). In China, the genetic relationship of sweetpotato has a faint correlation with the breeding institution but not with the region or flesh color, and they have not differentiated into obvious groups (Fig. 3 and Fig. S2). This may be because China is not an origin of sweetpotato, which was imported in the 16th century, and most of the present varieties or their parents were derived from the Japanese variety “Okinawa 100” or the American variety “Nancy Hall” [4, 33, 34]. In addition, the crossing of sweetpotato from different regions was frequent after the middle of the last century, so they have not been significantly differentiated. The molecular ID for each germplasm resources was constructed based on high-density SNPs and InDels, which could solve issues with distinguishing germplasm resources, such as one variety having various names and clarifying the origins of several landraces.

The highest peaks on Iba_chr5a were associated with the three anthocyanin-related traits (Fig. 4A–F). This indicated that this locus contained a dominant gene for anthocyanin accumulation. Previous studies have demonstrated that *IbMYB1* is the major gene controlling anthocyanin accumulation in sweetpotato tuberous roots [20–23]. The mapping positions of *IbMYB1* differed, which was the result of the use of different reference genomes from two varieties of *I. trifida*. However, our reference genome only contained the flanking sequence of *IbMYB1* (*IbMYB1-2null*) at this site, because of the following: i) previous studies suggested that the variations in the flanking sequence but not in the coding sequence of *IbMYB1* led to anthocyanin content changes in sweetpotato [20]; ii) only one set of subgenomes of Xushu 18 was used as a reference in this study, which does not contain the *IbMYB1* coding sequence, and thus the association occurred in the flanking sequence; and iii) reference variety Xushu18 was white-fleshed, and it may not contain the functional gene of *IbMYB1*.

The metabolic pathway of carotenoids in plants has been relatively clear and is composed of multiple enzymatic reactions. These enzymes are encoded by multiple homologous genes in plants, and they play specific roles in different tissues and stages of growth in plants [24, 25]. Among them, it is unclear which are involved in carotenoid accumulation in sweetpotato tuberous roots and which are responsible for flesh color in sweetpotato. There have been few studies regarding this. Diverse approaches and populations have been used to mine genetic loci associated with carotenoid content in sweetpotato tuberous roots, and some quantitative trait loci (QTLs) were obtained [35, 36]. Using a biparental mapping population, Gemenet et al. [37] mapped two significant QTLs containing the *Phytoene synthase* (*IbPSY*) and *Orange* (*IbOr*) genes. These two genes have been regarded as the major genes controlling carotenoid content in sweetpotato tuberous roots [37, 38]. In addition, other genes encoding enzymes were mapped [7], but no QTLs containing the *ZEP* gene were found. Through forward genetics (GWAS), we demonstrated that *IbZEP1* may be the major gene controlling flesh color (white or orange) by regulating the carotenoid content in tuberous roots (Fig. 4I). Expression analysis of *IbZEP1* may indicate that phenotypic differences are more likely to be caused by differential expression than by structural variations (Fig. 5C). Transcriptome analysis demonstrated that the *ZEP* paralog was a key gene involved in carotenoid accumulation in yellow-fleshed sweetpotato, which was consistent with our results [39]. ZEP catalyzes the conversion of zeaxanthin to violaxanthin, a key reaction for ABA biosynthesis and the xanthophyll cycle. In *Arabidopsis thaliana*, ZEP is encoded by a single nuclear gene (*ABA1*, *At5g67030*) [40], and it potentiates carotenoid degradation in maturing seed [41]. The *ZEP* mutant could change the color of plant tissues such as potato tubers [42], pepper fruit [43], rape flowers [44], and tomato fruit [45]. Together, these results demonstrate the reliability of the GWAS results.

Leaf shape in sweetpotato is a relevant agronomic trait, and several loci for leaf shape have been identified. Gupta et al. [46] conducted RNA-seq to explore the genes controlling leaf shape, and the expression of some genes correlated with the phenotype. Chen et al. [47] detected a locus for leaf shape, and *IbFBW2* encoding an F-box protein was considered the candidate. *YABBY* was not mentioned in the above studies, which were considered a candidate in our study (Fig. 4K and M, Table 1), thus indicating that *IbYABBY1* is a novel gene regulating leaf shape in sweetpotato. YABBY is a transcription factor unique to seed plants and plays a key role in the development of leaves and leaf-derived organs, such as cotyledons and flowers [26]. The YABBY protein is typically characterized by the presence of a C_2_C_2_ zinc finger structure at the N-terminus and a YABBY domain at the C-terminus [29]. By inhibiting the expression of the *KNOTTED1-like* (*KNOX*) gene, YABBY promotes leaf primordium production [26]. Mutations in *YABBY* in *Arabidopsis* resulted in radialized leaves, a phenotype very similar to lobed leaves in sweetpotato [26, 27]. Therefore, this gene is presumed to be involved in leaf shape in sweetpotato.

GWAS for autopolyploids is extremely challenging due to its abundant homologous sequences. Unlike allopolyploids, such as wheat [48] or cotton [49], in which the variants can be accurately mapped to specific subgenomes, the variants cannot be phased by present algorithms in sweetpotato, affecting the GWAS results. Therefore, GWAS in sweetpotato started relatively late, with few achievements, and it was difficult to obtain candidate genes [12, 50–53]. In this study, we attempted to identify variations by considering a set of subgenomes as a reference and ignoring the dose effect of each variant. Based on the results, this method was feasible to a certain extent. Of course, some real loci may be missed based on this method, and it requires longer reads, newer algorithms, and more accurate reference genomes to solve these issues. The genes obtained in this study also need to be further verified using transgenics. The GWAS-based specific structure of the phenotypic traits is also challenging, which may lead to false positives. To control false positives, two strategies were used in this study: first, two models were conducted, and only common loci were selected; second, multiple related phenotypes were used for GWAS, including three anthocyanin-related phenotypes (AN, FP, and SC), three carotenoid-related phenotypes (CA, BC, and FO), and two leaf shape-related phenotypes (LS and LLN).

In general, 314 sweetpotato germplasm resources were collected and re-sequenced in this study, and the genome-wide variants were obtained using a high-quality genome as a reference. Based on these variants, the population structure and relationship between sweetpotato germplasm resources were elucidated, and an excellent core marker set was constructed. As many as 20 agronomic traits of these germplasm resources were measured, and GWAS on these traits was conducted to identify several novel loci with high confidence, including *IbZEP1* and *IbYABBY1*. These loci and candidate genes will be further studied in the future. The above studies will be valuable for genomics research and genetic breeding in sweetpotato.

## Materials and methods

### Materials and resequencing

The association panel was composed of 314 germplasm resources, all of which were collected and conserved in the National Sweetpotato Genebank in Xuzhou, China (117.30°E, 34.28°N), and all germplasm resources were available. Fresh leaves of these germplasm resources were sampled and ground into powder using liquid nitrogen. About 700 ng of qualified DNA for each sample was collected. The NEB Next® Ultra DNA Library Prep Kit was used (NEB, USA) to construct the library, and index codes were added to the sequences of each sample. In short, the DNA was purified using the AMPure XP system (Beckman Coulter, Beverly, USA). After adenylation of the 3′-terminal of the DNA fragment, the NEB Next Adaptor with hairpin loop structure was ligated for hybridization. DNA fragments with a specified length were selected through electrophoresis, and the reaction was conducted under the USER enzyme (NEB, USA) at 37°C for 15 min and 95°C for 5 min. Phusion high-fidelity DNA polymerase, Universal PCR primer, and Index (X) primer were used for PCR. PCR products were purified (AMPure XP system), and library quality was evaluated using the Agilent Bioanalyzer 2100 system. A qualified library was used for sequencing on the NovaSeq 6000 platform. The insert was 350 bp, and 150 bp paired-end sequencing was generated.

### Reads alignment, variant calling, and annotation

All sequenced reads for each sample were mapped to one set (Iba_chr01a–Iba_chr15a) of the reference genome (upcoming released) using Bowtie2 [54]. Samtools [55] was used to remove low-mapping quality reads (MQ < 30), and the alignments were sorted according to mapping coordinates. PCR duplicates were removed using Picard 2.24.0 (https://broadinstitute.github.io/picard/). HaplotypeCaller and CombineGVCFs modules of GATK4.0 [56] were used to call SNPs and InDels, and the VariantFiltration module was selected to filter low-quality variants with the following criteria: for SNP filtering, QD < 2.0, FS > 60.0, MQ < 40.0, MQRankSum < −12.5, and ReadPosRankSum < −8.0; for InDel filtering, QD < 2.0, FS > 200.0, and ReadPosRankSum < −20.0. For GWAS, the raw SNP set was further filtered using Vcftools [57] with the following parameters: —max-missing 0.5 —mac 3 —minQ 30 —maf 0.05 —minDP 3, and finally filtered using PLINK [58] by LD as following parameters: —biallelic-only —indep-pairwise 100 kb 1 0.8. The output SNP set was used to conduct GWAS. The genetic variants were annotated using ANNOVAR [59]. Transcription binding sites were predicted using PlantPAN 3.0 [60].

### Development and verification of InDel marker primers

Plink was used to filter raw InDels with a heterozygosity of more than 80% or MAF of less than 0.2. The InDels with base numbers differing by more than five were retained. The sequences in the 200 bp upstream and downstream of these InDels were extracted as templates to design the amplification primers. Multiple primer pairs were simultaneously designed using Primer3 [61]. The parameters were set as follows: lengths of products, from 100 bp to 200 bp; Tm scores, from 58 to 64°C; differences in Tm scores between forward and reverse primers, less than 2°C; GC content, from 35 to 65%; and lengths of primers distributed between 20 and 26 bp. All primer sequences produced using Primer3 were aligned to the reference genome, and the primers that matched a location other than the target sequence or their alleles were discarded.

Eight sweetpotato samples and 30 primer pairs were selected randomly for PCR and polyacrylamide gel electrophoresis (PAGE). The samples were as follows: Fushu24, Xushu31, Chuanshu221, Zhanshu12, Yanshu25, Eshu11, Beijing553, and Liaoshu5. The primers used are listed in Table S10.

### Phylogenetic tree construction, PCA, population structure and *F_st_* analysis

The SNP set was used to construct the phylogenetic tree using Fasttree [62] with the maximum likelihood method (ML), and visualization was performed using Figtree 1.4.3 (http://tree.bio.ed.ac.uk/software/figtree/). The population structures were assessed using ADMIXTURE [63] with a CV error from K = 2 to K = 7. Visualization was conducted using the R package Pophelper [64]. Plink was used to calculate PCA with default parameters, and rMVP [65] was used for visualization. The phylogenetic tree for homologous candidate genes was constructed using MAGA-X with the neighbor-joining method [66]. *F_st_* was estimated using vcftools [57].

### Phenotyping

The 314 germplasm resources were planted in four cities in China: Wenchang city (110.80°E, 19.54°N) in 2019, Xuzhou city (117.31°E, 34.27°N) in 2020 and 2021, Jinan city (116.85°E, 36.35°N) in 2020, and Tongliao city (122.26°E, 43.62°N) in 2021. All qualitative traits or pseudo-qualitative traits were investigated, and samples showing inconsistent results at two locations were discarded. The phenotype values were coded for GWAS (Table S11). To measure the content of dry matter, three fresh sweetpotatoes were cut into pieces and mixed; 50-g samples were taken and dried to a constant weight in a freeze dryer, and the dry weight was recorded. The dried samples were broken into powder using a high-speed blender, and the powders were screened using a 100-mesh sieve. A D-Glucose Assay Kit (Megazyme) was used to determine the total starch and soluble sugar content in the sweetpotato powder. Reducing sugar and crude protein content were determined using near-infrared spectroscopy (NIRS). The total carotenoid and total anthocyanin content were determined using the colorimetric method. The β-carotene content in tuberous roots was determined using high-performance liquid chromatography-mass spectrometry (HPLC-MS). Three fresh sweetpotatoes were cut into pieces and then mixed, and 6 g of them were taken and mixed with 40 mL extracting solution (acetone: methanol: formic acid = 25: 75: 1, volume ratio). The samples were centrifuged for 20 min (6000 r/min, 4°C). The supernatant was concentrated to 2 mL for testing on HPLC-MS. β-carotene was used as an internal standard. The contents were calculated according to the standard curve equation Y = 4218.124X − 1877.159 (Y is the strength value, X is the sample concentration). For the weight of tuberous roots per plant, at least five plants were investigated, and the mean values were recorded.

### GWAS, gene expression analysis and amplification of *IbMYB1*

Association analysis was performed using GLM and MLM by rMVP, and the significance threshold was about *P* = 10^−8^. The loci were detected with both models, and obvious peaks were considered to have high confidence. The regions 200 kb upstream and downstream of the peak SNPs were scanned, and candidate genes were selected referring to genome annotation. The correlation between phenotypes and SNPs in candidate genes was evaluated and drawn using OriginPro 2020b (https://www.originlab.com/).

Gene expression analysis was conducted using qRT-PCR. Total RNA was extracted from tuberous roots (*IbZEP1*) and leaves (*IbYABBY1*) using an RNAprep Pure Plant Plus Kit (Cat. #DP441 and Cat. #DP432, respectively) by TIANGEN Biotech (Beijing) CO., LTD. cDNA was synthesized from total RNA using a PrimeScript™ II 1st Strand cDNA Synthesis Kit (Cat. #6210A) by Takara Bio Inc. (Shiga, Japan). qRT-PCR was conducted using the SYBR® Green Realtime PCR Master Mix by Toyobo Co., Ltd. The following primers were used for amplification (5′—3′): for *IbZEP1*, forward, TGACGAGAGTTATCAGCCGC, reverse, GATCACCTTCGTGTTGCTGC; for *IbYABBY1*, forward, TGCACTACCATTCTTGCGGT, reverse, ACAGTGCCCACATCTGACTG. *IbARF* was used as a reference gene to normalize the gene expression of targets [67]. The ABI QuantStudio 6 Flex system was used for PCR and to detect the fluorescence signals. The samples (1–10) for *IbZEP1* expression analysis were A28, A30, BZ69, A24, A27, A47, A8, A53, A54, and A10 consecutively. The samples [1–10] for *IbYABBY1* expression analysis were A2, A4, A23, A24, AH33, BZ41, BH24, BH20, BH57, and BZ29 consecutively (Table S1).

Two pairs of primers were used to confirm the replacement of coding sequence of *IbMYB1* (5′—3′): P1-F, TGCTAGGCTCTTCTATGCTCC; P1-R, TCGACTTGAGAGGTTGTGCC; P2-F, CATAAACGCTGCTCAACGGC; P2-R, GGCGATCGTTTTGCTTGTGT. The DNA template was extracted from leaves of “Xushu18”.

## Acknowledgements

This work was supported by the National Key Research & Development Program of China (2018YFD1000705/2018YFD1000700), the Natural Science Foundation of Jiangsu Province of China (BK20221213), and the China Agriculture Research System (CARS-10-GW01). We thank LetPub (www.letpub.com) for its linguistic assistance during the preparation of this manuscript.

## Author contributions

Q.C. and S.X. designed the study. S.X. conducted the data analysis and wrote the manuscript. X.D. prepared materials for genome resequencing and investigated aboveground agronomic traits. Li.Z. measured most physiological traits. Lu.Z. verified the molecular markers by electrophoresis. Z.Z., B.G., A.Z., D.Z., R.Y., J.W., Y.W., and Q.L. participated in the planting, phenotyping, reaping, storage, and transport of plant materials. P.X. assisted with data analysis. Q.C. supervised the research and the manuscript. All authors read and approved the manuscript.

## Data availability

The sequence read archives of 314 samples were deposited in the NCBI database (Accession No. PRJNA857483). The SNP dataset and the phenotype data were deposited in a publicly available database (https://zenodo.org/) and can be accessed via the DOI number (10.5281/zenodo.7184909).

## Conflict of interest

The authors declare that they have no conflicts of interest.

## Supplementary data

Supplementary data is available at *Horticulture Research* online.

## Supplementary Material

Web_Material_uhac234
