# Supplementary material for: Resequencing of sweetpotato germplasm resources reveals key loci associated with multiple agronomic traits
Source: Hortic Res. 2022 Oct 19;10(1):uhac234. doi: 10.1093/hr/uhac234 (PMC9832839; doi:10.1093/hr/uhac234)
Supplement: Web_Material_uhac234 [file web_material_uhac234.zip › Table S8.docx]

**Table S8 The haplotypes of candidate genes in reference genome**

>*IbMYB1-null,* 5’-3’

TATTGCTCTCAATGTGCAAGAATCAAATGAAGTATCAATGAGAAGTTTAATTGGGACATATTTATTGGTACCAAATAGAAACAAACTTGTTAACTCTATTACTAAGTCTTTGACCGTTTGCCTACGAACCTAGTATCAATGGGAGTTTCTCTCATACATTTGATAAAATAAAGAGTAATGATATTTTTCCCTAAAAGAATTCTTCTCAAAATTTTGTGTGACATTATTTAATTGGCCACTTTATTTTTTATTTTTTTCATTAAGGGTCTGTCTGGAAAACAGGAAAATATCTTCTGAAAAATGAATAGAATTGTATAATTAAACATTTTAATTATTTTATTTAAAATATAAAAATATTTATAATAATATTTAAAATATGTTATTTATTATTATTTTTTAAAATGATGTTTCCGGCGGACTGGTTTCGGTCAGAAACCAGTCCGCCGAAAATGAGAGCCTAGGTTCAGAAAATGTAAAGATTTTTCTCTGTCAACGGAAAATGTTTTTCGTTGACTGGATTTTCCAAGCGCATCCAAAATGTAAAGAAATCATTTTTCGAGTTCCCAAAACCCTAAATGTGTGTTGATGTGTAGTCAGCTAATTCATTGCACCCAATGATTATAAAATATGTCAGGGAGAGAATTTAAGAGAGAAAAAATTAGAATGAGTAAGAAGCATTTTTTCAATAAATAATTAAGGGTGTGTTTGGTTGGTGGGTTTAGGCATAAGGTATGTGTATGAAAGTGATTATTATTGTTTGGTTGATAGACTTTTAGAATACTACTATGGATTTGAAATACCCTATTAATTGAAAAATTCATACCCTAATGAAATAAGAGTTTCATTCTCCTTTCTCCTTTCTTCCCCAATTATTAATAATCATTCCCATTCCACCATACTATCAAACATGCAAAATACTTTCACCAAAACTCATTACCATTACCAAGTATTTGATACTCATTCCGATTCCAAATCCCATGTGCGAACCAAACACCCCCTAAATAAATAAAAGATTCGTATGTAATGACAAATTATTGGGCAATTAAGATATTGCTTAAATTATATAATTTTTACAGAATATTATAATACTCATCCAACGGTTCTCGATAGAGGCATACAAGTCGTGCTCTAGATATTTAGAAACATTTGGAGCAAATCCAATGATTTGACACAGCAAATATTTGTGTGGCCCACAAAATTTTTTTATGCACCTCAAAAATTTAATGATGTCTAATATAATGCATTAGTTAATTTCTTACTTATTAGTTATTACATCAAGTTAAATTAATACGATTTGTATAAAATGACAATCATATTTATTACATCAAGTTATTTTTCAAAAAAAAAATACAACAACAACAACAACAATAATAATAATAATAATAGTCAATGACAATTTAGTTATTTTTCTTAAATTTTTTCTTTTTTCAAGATGCATTTCATTCCAATTTCTAAGAGATATATGAATTGCAATTTCGTAAATAGAGAAAATTACAATTTCACTGAATTTCAATTCCATCCATCCAAACACTGTAGTTTATAGCTCCGTTAAATTATATTGTAGTTAAATTGAAATTTATGTCAACCAATTAACGGAACACTCTCTAAGGGATTCTCTTGTAATCTAAAAAAATGAATTATCAAAAATTTAAATGTTGTATTTAAACCTGTCTTATTCACAAACTTTATGTGATCATACAGAATCTACATAATGATTTTAATAAAAAAAAATTAAGAAAACAAGTGGATTTCAAAAAAAAAAAAAAAAAAAAAAGGGGAAAACATGTGCAGTGTCGTCATGTAAGTACTACAATTATGGATTTGCTTATAATAAAGTCAATACCTAACAATGTCTCTCTTTCACTCTTCAAATATGTATAGGTTATAGCTTTGCTAGGCTCTTCTATGCTCCACTAATATAATTTTGAAATTATTTAATTTAATATATATATATATATATATATATATATATATATATATATAAATCTATTTAGGTGAGAACTAGAATATTATAGAGGTCTATGAGGTCAAATAGTGATCGTTAGATTAAGATTTTGGATGGTAAGATTGATGTTATTTTTTTAAAGTTATTTATTGATTTGGGCGATGGTTTTAGAAGCCTGGGGGTATTTGGGTCCTTTTATTCAAATGTTTTAATTATGGTAGATTTCAAACTTTGTTTAATTTTGGTATTAATCAGTCAGTTTCTTTTTATTTACCTGATTGTTGGGATTCTTTCCGCTCTGTCCGAGGGATTGTGTTTTCGACGGTGGCTCTTCAACTGCTCATCGCCTCAGTTTCGACTTTCAATTTTCTTCACTCCACTGTTGGTGACCGGCGACTGGCGCAGTTGAGAGGGACGTCGGCGACTACGTTTAGGCACAACCTCTCAAGTCGACGGCCTCCTCTTTCCACCTACGCCTCTTCATTTCCTCCGCGACTACGTTTCCCCAACAAACCTTGCCTCATCTCCTCCACTATTCCCAGGTTTAATTAAGTTTCTTCTCATCATTATCATATCATAAATTTTTGTGTGAATATATATGAATTCTGCTAATATGTAGGCAATGGCGTTGAGAGAAATCGAACAAGAATTAGAATACATTCATTTGTAAATTTTCTGTAATGTTGGGTTTGGTTTGGATTATAGGACAAGAGGGAGTTCCAGTTGGAACTTGAGAAACCAGTTAGGCATGTGCCATGGTGCAGTGTGAATCCTTACCCTTCACCTGATTCAGAAGAGAAAGAAAAAGCAGAGTTTCTTCTATATTTCAATGGGCCTAAGCCATTTGAAGTTACAGTAAAGGCTGGAGAGGTTCTTTATTTGTAAGCATTTCCTAAATTCTCTCTTATCAATTCTAATGAGAATTGACTACTTAGGTTTGTTAGGTTTGTTTAGTTACATGTTCTTAACACCATAATCATTTGTTGTCTGTAACTTTGCTCAATTGTATTAGCTTTTCTCGGTTAGATATCTGATCTCTTAACATAGACATGTTTGCAAAATCCTCCAGGCTGTGGTAATAAAGGATACAACTCTTGGCAGTGGAAAAATTTCAAAACTTGTTTTAATTTGCTAAAGAAAGGGTTATGAATACAAATACCAGTTAGGTTGTAGGGTTTCGGTTTTTTTTTTTTTTTTTTAAATTATTCGGTGTCCCGGGAGTGTAAATGACGTTTTATGTGTGCCAACATCCTAATTTCACTGAAATTTTGTTAAATTACTGAAAGTTTGGCATTACAATTTTTTTTTTGTCAACATTCTTTATTGTCAAAAGGCTTTCCTGGACTCTGGCTTGCCTTTGGATAATATTCATAATATTGAGCTTGTCAGGTCCAGCTTTTGGCCACTGTTTGTCACATTGTGTGCCAGTGTTGGTTATAATGTGTGCCACTGTTGGTATAATGTGTGCCACTGTTGGTATAATGTGTGCCACTGCCTAATTCAGTTGGGGGGAATTGAGAGAGATGGAACTCCTATTTTTGTCTGCCTTTGTTGCCTGTAATGTGTGTCGTCACTGTTGTTACTTTGAATCAGATTGTGCAAAATTGTATGTTTGTAATTAGTTATGAATAATTGTAATCAATTATTAGTGTGGTTGGAATTTTTAATAATTCTTTAATATCTTGGGATTCTTTAATATTTTGGTATTGTGTGCCATTCTTTGCTATCCATTGTTGTTCTGTTCGTCTGAGCACACAATATATTAAAATATGGCACGCATTGTATCAGAATGTGACACCCAATATAGAATAACAATGTTTCGCGGATCATATATATCATTTTTTTAAAAAAAAAAAAAGAAGACGTAAAGTGATAATCATTTAAATTGCACTCAATCAATCAATAATTATTTAAATATATATTGCTTCAAAAGAGATGAGCTAAGTGAAACATACTTCTTTATTATCAATTTAGTTCGGAATCTGTCATATGCGATTTTTTCTAATGAAAATCAATATGATTCAACGTAACATAACTGTGATTAGAATTGGGGGGTCCGAGTGTATTTCCACTGTTCATTTCATTATCCTAAAAGCTCTGCTTGGGCGTTTTTTTCAATTTAAAATTTCATCATATAAAAAAATAGTTTCTATTCTTGGTATTTTGACCGTTCTGATGTGGGCACACACCATATGTAACTCGGGCACACAAAAACACCGAACATGGCACGCGTTTTCTTAGAGTTTGCTCTAGTATATACATGCGGTATTCAACGGTAGACAAACAACTAACACATGGCTCACACAAAAATACACATTCGTTTCATTATTCTAAAATATTTGAATATTTAAATACGGTATTTCAACACTTTCTTATATTCATAATTCAAAAAACTCTTGATTGGGCTTTTTTTTCAATTAAAAATTTCGTAGTATAAATAAACAGTTTTTGTTCGCAGTATTCTGTCCGTTCTGATGTGGGCACACCGAGCCATACACCCAACCCTGTTTGACACACGTCGGCGTGTGTTTATATTTGCTCAGTTGGCCTCCCCTGTGGTTGTTGACCTTTGTTCCTTTGTCACTGTGTACCTCTCTCTTGTACAAAGTAAATACTTCACTTCTACATCTCCATATCGGTTCTTCTTCATTGTGCTGCTCCGGACATCGGAACCTACAAATGCGGCATACTCTTTGTAGTATCGAATTGAACACCTTCGTCTAACGTCGAAAAACGTTGCCCCAAGAAGGGTTTAATACTTTCATCACAATCAGGTATACATTCGTATGCCCCATGTTCTTCATTGTCGCACTGTTCACCCACACGCGCGTCACCGTTGATGTTTTCTTCATCCGACACACATTTAATATAATTACGGCACATACAGTTGCTCATTCTGTAACTACTTAAAATTAAACAAAACAATCTTCTGAAAGTTAACAAACTATGTCGGGAATATTAAAATCAACAACAACAAATAGTTCAATATGTTTGACTTCGTGAATCATAAAATATGTCATCGTTGCATGCAGAAAACACCATACAAAAAAACACATTCATTTCGTTGTACGATATCAACTGCATATATTTCAGTAGTCAAACACAATTCACTGAAACGACACACATTAAACCCTAAGATGGCACACAATTGAAATATAAGTAATATTTTCCACTCATTTTCTTCCCACCCACATGAAATTTCTTTCGCTTTTTCTTAAAACACAGCAAGGCACATACAGTGAACACTTATACTTTATTCAATTGCATACTTATTATCCAATTAGATTACAAACACTCAAAAAACACAAGGAAATCAAAACTAAGTAAATCACGCAAATATGTACTCAAAAATTACAACAATCCTTGTAATTATTCGAAAAAATATGTAGTAAATCATGAAGCACAATATTCTCACAAGGCAAACAATACATAGTGAAAAGTCACATAAACGCTGCTCAACGGCACACAAATTTTCAGGCCAAAGAAACCAATCCATCATATCCACATTCGAATGCTTAAAAATTATTAAAAAAAAAAACAAATTGGACTTTGAGCATTAGGCACATGTATGCATGGTAACACAAGCAATGTGTATAACATTAAAGTATACCAATCTTCAAACAATGTCATGTTCGAAAATACTCAAATTACTAACTATATTCCTCACTATATACCATAGAGTAGTCAAACACAATTAAGGGTAACGTCACATCTATTCCCCTAGTTGTCACACAACGAATACAGGCATCATGTCTGAAATTTTTAGGATATTCAACCCATATTCCAGCATCTTAGCACGTTGAAAATACTGGATATACATGTTCCTTTAATCTCACCCTCATTCCATAATTAAAAAGATCATCAAATGCTAACTTTGTAAATAAAAATCATACCTTAATTAATACAAAATAAAAAAACTCTAATTTACCCCTATTTGATCTCAATATGGCGTGGACAATTAAATCTAATCTTTAATCTAAGCCTTTAAATCCTTAATATTGGATGGTTGAGATTACACCTCTTTTGATCTCAAAATAAGTTGTCCTCACCTGAATAGGAGCCTATATATATATATATATATATATATATATATATATATATTTGGCTTTATATTAACGCACCAATAATGGGTTGCTATTTTGAGCAAACATTTTCTTACTACTTTTAGTACGACATAATCCTCAGTGACTGTATCCATGCGACATTTACTTCCCAATTTAGTTTCCATTCATGTAAATATTAAATTAAACTAACCAAACACATATAAATTAGATAAGATATAGCCCAACATTCACCTGACAACATTGAAATTGAAATCTTTTTGTCTAACTTAATTAAAGTGTCGCATTATAATTACTTTTTTTTTTATCTTGTGTATAATAATAATAATAATAATAATAATAGGAAATTGGTCAACACTTTACTCAAGAAGTCAATTAGACCCTTAAACTTTTAAAGGTTGCGATTAAATCCTTAAACATATTATTTTAGAGCATTGAGACCCAAAAATCGGTTGATTACCTGTAACTACAGGTCATTAGAGTTCTGGCCAACCTACGACCATATTCCGACCAAAAACACAAGCAAAACGATCGCCGGTGTTGGTTAGTGTAATGACACATCTATTACCATTCTAAATGAGTAGTCACAGTATATTACCTTGTACAGAATTGAGTTTTTAAAAAAAAGTTTCTTTTAATTTTTTTTAAAAGAAAAAATGGTCAAATGGGCTCTTCAATCTTACTTAGGAAGTAATTT

Note: the sequence marked in yellow is 5’-flanking, the sequence marked in grey is 3’-flanking and the sequence in between is unknown.

>*IbZEP1,* 5’-3’

TGACTTGTAAAATGGAACCGGTCCATGCATAACAACTGATTCCTATGTACTATTCTTTCTCCAAACTTTGGCTCCATACTTCGAGCTTTACGAGATTTTCTTCTAGGCCTCAATCCACTCTGATCTAGTAATAGTAGCCCAAGATTGGCCCATACTGAAAATCTTTTCATTGATAATCCATTCAATAATTTTTTCTTTTTAACTAAATTAAATAGCTAATAGTGACCAAATAAGCTAAAATTGACTGATAGACTAACTATTTTACCAAACAGGCTATAGTCATTTAGTTTGATTACATACACACACCGGCTCCAGCATGGGGGCAGCTGCCCCACTCACCCCACATCCACCCCTTATATAGTCTTTGTATGTATATATGTATCTATAGTACATGTTTTAGTATACAAATTTAAAAACATATACAATTAACAATGGATCCGCTAGACTAGGTGTACTTAACAGAAATACGTAAATAGATAAATGTAAACAAGAGATTAAGTTGACTAGTTGGTAGATGTGTGTTTGTGCTAAGAAATGTTAGAGTTCAATTCCTTATTCCTTCAACAATATGTATATATAAAGTTCCACACGCCCCAATGGGTCGAAATCGGCCACAATGAACTTAACAATAGTTGGTAAGAAAGTGCTATTTGAATGCATGTAAGAGAATTGATAGTAAAGATTAACGTGTGCAAGCCATAAAAGAAAAGATGACTACATAAGATTTGAAGACATGTAAAAGAGTAAATAACCTACAAACTTAATAGTGCTGCTCCTTAATATTCTTGATTTAAGCGAGTTAATTATGATTAATTTAGACTCAAATGTTTCAAATTTCACCAACCGCTCTCCCAAACAATGTCTTTGATCAACTAACATTAACAAAAGAGTAATTTGTGGTGTGTGTTTTTCTTTTGAAGAGGATGGATATAATTCGCTACCAATAACTTGAAGAAACAAAACTAGGATTCATTGAATAGTTATATGTACAGGTGTTGGTAGAGGCGTGTGTTGGGTGAAGAAGACATATGGACATGTGGACATATAGCTTTTTTTTTTTTTTTGAATGCTACTGTACTTTCTCAACCTACTGAAGAACAAAGAGACAGCAACGCCTCCACTGAGGCTCGAACCCACCACCTCCCATATATAGGGAAGGGTTTGATGCCACTGGACCACAAGGCCCTTGGCAGGTGGAGAGCTTTTGAGAGTGGAGAGTACTCTCCAAAGTAGTACTCACCCTACGCCGCTCACCTTCTATTGTTACAGTCAAAGACCATGATTCATATTACCTTGTGAATTAATACATTTCGTATCACTTTTTATACAAAATAAATATTTAACTGCAGGTACTAATGTTATTTTAGATCAGGATTTACAATGAATGGTAAATCCTGATTCCTGATATAATTTGCCGCTGATCTTCCCTCATTTCCTCACTCACTAGTCATTACCAGATTACCTTAATAAATCCCAAATTCAAGACACGATATTCTTGAATTCACTGACACTCAAAGTATTTATACACGGGAAAGATCGAAATTTGGACTTTCTTTTTCATGAAAGCACCAAACACATTGACATTAATTGTCACATTATCAAGAAAAAACTAAATCAAAGGCGGATCAAGCACTTGTCCATATCATCCTCAAACTTGATAACAGATAGATTTACAAAGCCGCTACCAAACTCCGAGTTTAGTCAGTTCATATCTAAGCTAGGCATACAAGATTTGCATGCTCTAGCTTAAGGGGCATTGAAGAATAGTAGCTAAGGGCATTCATGTAACTTCCATATATATCTCATGTTAGTTGCAACCGTCATCTACCCACATTTATATTTGCCATGTATGTACATGTTGTAAGCATGAAAAACACAATACAAACTTTCCCTTTAACTTTCTTCTCTGTTCGCTCCTTCCCTCTCTCCGCATCACTGACCCTCAAAGCATTTATACAAGGGAAAGATCGAAACTTGGGCTTTCTTTTTCCAGGAAAAAATGAGTTCATCTGTATTATTGCACACTTCAATTCATACCTCAAGTACAACAGTGAAGGCTACACTATCATCTTCTGGCGGGGACTCAAAGAGGAAGCTGAAGATACTGGTGGCTGGTGGCGGGGTTGGAGGTCTGGTTTTCGCGTTGGCGGCGAGGAAGAAGGGGTTTGAGGTGGTGGTTTTTGAGAAGGATTTGAGTGCTATCAGAGGGGAGGTGTTAGACATGTATGCCCTAGGAGTCAACATTTATTGTTTTGGGCATTAATTTTTTATTAAATAAATATAATAAATAATTTATTTATTTATTTTTATTAAGGCTTAGTTAATATTTGATATTATTCCTTACAATCTTCTTAATTCTTCATTCTTAAGTGTTAAGAATATGAGTGACGAAGAATTAATAAATGAAGTATTGTAAAAATGTTCCTAGTTATAGGAATTCTAATTGGGCATTAGAATTCCGATACGACTAGCACATTGTCCTTCTTATGGTGAGTCTCATGCCATTCTGTATGAGATACAGAGAGTGGACATGTGGATGATTGTTAGAGAACAAGTCATTGAACATTGACTGACTACAACGTACCGCATGGTGTTTACCTACGTGTCATCGGTATGTTGTATGTTACAAGTATGCAATAATCCTTTGACTTGAGACGACATGGTTGTCTCGTACATATGGTGGACTAGTTTTTGCCAGTATGCGTCTTTTGTTCCTTATGGGACTATAAGTATACTTGGTGGCCTAGTTCAGTATTGTACGAAGACATGTGTGCGTTCAATAGAGGATCCACCGCCTTGAGGTAACGAGGATGTTCCAGTCTATTCAATAATCATACAACTAGAATCTCTGGGCAGAGTATAATGAAGTTGGACTTCAGGTTTAAGAATATTGAATAGACATTGACCGGGTTTATAGGTTTGACCAAGTTTGACCATGACCTTTACCTGGTTCGGAACAAGTGTTGTGTGGAAGGAATGTTGCACAATATTTGTAACGGAAAGGTTCATTATAATATATTAAAATATATTCATTGTCAACTAGGTAGTCATGACATATTGCTAGGTGTCACTCATGACTTACGAATTATTTAATAATGAGTTATTAAATAATAATCGTTGCTAGTTTGACTATGAACCTAGAAAGTCACACCTTAATGGTTCGTGGTAATGCTGAGAACTTTAAAAGAAATCGATAAAGAGTTATCGATATTGAATTAATACATTAATAATTGATTAATGGATTAATTTAATATTGGGTAGTTTTGGGCTTTTAAAGGCTAGCACCTTTGGGCCCAAACCAGGCTATATATATTGCCTTGCCCAATTTCTTAAGGGATGAGAGAAACACAATACAGAGATTAGGAAAATAGTTTTCACGTAGAAATCTCTGGGAGTTCTCGCGTGCCCGACCGGTGGACAGACTAGAGGCCGGACGATTGGACGGTTTTGATTCTGCCAAAGCACGCTTCAAGGGTAAACCTTTATAACTCCCTCGTTTACATAGAATCATAGAAAAGCGATTTGATGCTTCCGTTGCGCATGTATGTTTTTCTAACAGGAGGatgtcaGTCAGTTCCGAGGTCCAATTCAGCTACAGAGCAATGCTTTGGCTGCTTTGGAGGCCATTGATATGGACGTTGCCGAGGAGATCATGACTGCTGGCTGCATCATTGGTGACAGAGTTAATGGTTTGGTTGATGGCGTTTCTGGCAACTGGTGAGCTCATTAGACTCTGTTTATAGGCCTTGTTACAAAAATCTGGCCTAAATGCTAATTAATCGCAGCCTAGGCGCCGGTCGACCGCCTAGCGTATCATCTTAAATGGTGGTCTAGGAGACCGACTATGTCGCTTAAGTACGGTTTAGGCTGCAAGTGCTGTCTAGGCCGCTTAGGAGCTGACCTCCTAGGTTTCCTAGGCGCTGACTAGACTTCCTAGGCACCGACTAGGCCGCATAGTCGGCCGATTAGCTATTGTTTTTTTTTAATGGGTTATTTCGCTCAAAACGATATTGTTTTGAGCTAAACGAAATAACCCTAAATTATACAACCTCTAGATATTTTTTAGGTTAATATTTAATATTTTAGTATTAATTATTAAAGTATTATATAGTTTATAATTATTAGTCTTTAAAAAACTTAAACAAATTTTTTAAAAAAATAAATAAATACAAGGGCCCCTAGGTGTCAATTAATCCCTTCGTACTACATGCGCCCAAGGAGCGTCTAGCGATTTTTTCAACCATGTTTATATCTTTAGATTTTCTGTGTTTTCTGCAACACAAAATCATATAACATTCCCCAGCCAAGTTCCTCGGGCCATAAAAGGTTTAACTCTTTCTTGACTGGTTTACTGGTTAGGGTCCGAAGATGGGTAGGTAATGACTGTGGGTTTTTCTCGTGAAATATATTATAATGATTGTTGTTTATCAGGTATGTCAAGCTTGATATATTCACTCCTGCAGCTGAACGGGGACTTCCGGTGACGAGAGTTATCAGCCGCATGGCTTTGCAACAGATCTTGGCATGTGCCATAGGAGAGGATACTATCATGAATAGAAGTAATGTGGTCAACTTCAAGGATGAAGGAAATAAGGTAGCATGAAACTTGAATTTTAGATTATGTTTTGTAGTCGAGAAAGTTTAAATTTTTCTGGAAATTTAAAAGCTAAAAAAACTATTCAAATGTTTGTATTAAAATGGGTCAATGGATCCTCGTAGAATTATATTCTTATCAAATACATTCATTAAAAATATTTTTTCTAAAAATTTAATTGATGAAAAAGTACCAAACGCTCTCTTAAAATTTTTCTTTGAGTGAGTTTTCCTGTCTCACGAATTTTTATCCATGAAGCAATGAGATTTTACCTTCTTCTTCACTCATTGGAGTTTAATTTAATTTGTTGTTTAGGTTACCGTTATTCTTGAGAATGGGCAGCAACACGAAGGTGATCTTCTAGTTGGTGCTGATGGAATATGGTCTAAGGTATTTGTCTCTTTCACCGATTTTTTTTAAATTTTATTTATGAAAAAACGTACGGTCACTAACTGACCAGCTCAAGACAAAAAAAGTCAATATACAATTTTCTTATGGAGTCAAACTCGGGAGTGGATTGCCCAACCTTATTGGACAAATTATACTATGGACTATTTTTAGTACGGAGTGCATTGAACGTTCATTTTTAATGTACTAAAAGCTTTTTTTTTTTTTTTTGGTAATATATCTAAAAATAAACTTTCAATACACAAAAAAATAAACATTCAATGTACTAATAATAAACCTTGTGTTAATGATTGGTCCAAAGTATATTGATTGACTTTGTAATTATTAAATCATTTGTCCGACTAATTCGGTTTGATTATCCCGTTTCACTGAATTTCTTGATTGTAGCTAGTTTCTTCATTCACAATATTTTTACATGGGCTTATGTACAAATACTAGGTGAGAGCTAATCTGTTTGGGCATAAGGACGCCACTTACTCAGGCTATACATGTTACACAGGAATTACAGATTTTGTTCCTGCTGATATTGAAACTGTGGGGTATATTCTCTTCTCATTTCTTTTGTTACTTTTATTTAGTCACCCCGTAACTTTTTTATACAGCATGAAACCAAAGATTAAAAAAAAAAAAAAAAAAGATGTTCAAAGTTTAGCCTGATCATTTCCTTTATCATTAACGGGTTGGCACGATAATGACATGTATAATTTCAAGTTCTTGTTAGCCTGTATATGGATTTATTTTAGACATTCTGTATTTTTTTTAAACATTTAAGGTGTGTTTGGTTGACATATTTAGCTATCAAAATCATTGTTATTATTTCTTAAATTTCTCTGATCTTGTGAAGGTACCGAGTGTTCCTAGGCCACAAACAATATTTTGTTTCTTGCGATATGGGCGGAGGAAAAATGCAGTGGTATGCTTTTCACAACGAATCAGCTGGTGGTGTAGATTTGCCAAATGGTAAAAAAGAAAGATTGCTAAAAATGTTTGGAGGTTGGTGTGACAATGTCATAAACTTATTAGTTGTTAGGGATGAAGATGCTATTTTTCGACGCGATATTTATGATAGAACGCCATCCTTGACTTGGAGCAAGGGTCGCGTTACCTTACTTGGGGACTCGATCCATGCTATGCAGCCAAATTTAGGTCAAGGTGGATGCATGGCCATAGAGGTATAAGTCCATCACTACAAGAAAAATAATTTTTAGTGACAAAAAATTCGTACGCTTTATCTTATTCTTAGCTCCTTTATTATCTCTTTGGAAATTTGGCAAACTCAATCAGGGAGTTTGAGTTGTTTTATATAATGTACATTCAGTACATGAATAATGTACTATTTGTTATGGGTGTGGACCATAATTTGCCAAATTTAATTATGCAGCTTCTTGGATCAATTCATGCCCTTTGGGCTAGATTTTGATTAGAGAAGTAGTGAAGGGGTTTTAAACTTTAGTCTGTATAATTGCAGGATGGGTATCAACTAGCTCTTGAGCTTGACAAAGCTCGGAGGCAAAGTGTTGAGTCGGGAACCCCTATAGATATTGCATCTTCATTAAAAAGGTAAATCAAATTTCCTAAGTTTGTTGACTAAAAGTGAAGCGTAGGATGAGTTCAAATGAGAACAATTCTTCATGTAAAAAATGAAGATAGATCACAAATTCACAATCCCATATAGGAGTTTATCAATAATTGAAATTTTAATTAAAAAAATACTGATTAAATTTTTAAGTTTGTAATACTTATTCATTAAACTTTTTTCTTTTAAAAAATTATTAAGCATTTAATAATTTAAACAAATTAATATTTTTTTTGAGGAAGTTAAACAAATTAATAAGTACAAGGAAAGTAAAAGAAATTAAACTAATATTGTATTATTATTTTAGTTTTAGAATTGAATTAAATTGTTAGTGAGGCTATTATGTAATTAATAGGTTATATCTTTAACCATAATGCATTTTCTTTTCAAATAATAATAAATTAACCGTATTTTGGTCATTCAAATGGATTTATATTATTCCAAACACATTTATTTTCCAATGGGGAAAAACGTTTTTTCAAATTAAAGCATTCCCAAATCTCATTTTTTTAACCCTATTAGTATATATATATATTTTGAGTACTATTGATTCTGTTACAATACAGTATTTATTTATAACTACTTTCTCAGACTCGAACTCACCCCGTACAACCGAGTGCTACTAGACCACAATGTCTTGCTATATTTTGAAGATCAATTTTCAGTCTCCTCCATTTATCAATTTCAAATATATGAATATACAAATTTTATTATCTCCACACTTAAAAAGAATCTAAATTCACTTTTTCTCCTTCTCTTCGTTTTTTCCCCAGTTATGAAAAAGAGAGAAGACTGAGAGTTGGAATCATTCATGGGCTGGCTCGAATGGCTGCACTTATGGCAACAACTTACAAGACATACTTAGATGTAGGACATGGTTCATTATCGGTATGAACTCCTACACCTCTTTTCTTCTTTTCTATTCTTATAATTTTTTTAATAGGGAGTTTTCATGCACTTATAATGAACTATATATTGTTTGTCTTGCAGTTCTTAACCAACTTTAGAATACCACATCCTGGAACAGTTGGTGGGAGATTTTTTATAGACATTGCGATGCCTTTATTGCTTAATTGGGTCCTAGTAGGCAATGGGTAGGATCTATATATGAATATCTTGGTATATATTTTAAAGCTAAGTCTCATTATGCATAGACAAAGACAATATAGGGAAAGATACTCTGGTGGGTGAAGTATGATTATCATACTTGAATGGTCATGGGTTCCCTCGTCAACCAAAAATATAGGGAAACATGCATTTTTTAATACGGTTACTCTATGCTAGTCATTTAAGTTCATTTTTCTTGTTGCTCTAGTAGTATTCCTGCTAACTTTTTAAATAAGTTACAATTTTAGTCATACGAGCTGAGCAATAGAGGTGGAAATAGGCCGATCAAGGCTCTCTAGCGCTTACAACTTGGCCTGATTTGGGTTCGCCCAATTGTCATATATATATGGACTACTATGTACTAAGTAAAACTTTAAGTTGTTGATAGCAT

Note: the sequences marked in grey are exons.

>*IbYABBY1*, 5’-3’

TGATTAATATAAACTAATCAAACAACCCTCTATTCGAGATTAACCTTTTATAATCATTTATGTGGATTTCGATCATAAAAAGCGATAAGAAAATATCTATAAAAAAAAATTACTAATGAAAAACTTTATTTTCTTATATTTGTATAGACCAACGCCAATACTATTATAGAACGTTGGACTCAACTCTCTGAGCGTCACACAATAAAATCTTTTTGGAATTGTTTATTTATTTAATTAGACAAACATATACAAGTTTAAACTAATTTAATTCATGAAAAAAATTTATAGACATTAAGTGTTACTAATTCCACCTAACTTATGAAAAAACAAAATAATAATTAGGTATATACCATACTAAAGAATAATTATCTAACTATGAGATCCTTATTTAAAAGAGTGTATTAAGATGGGAAAAGATCTTTACCCTTCTTCTTCACACCATCAAATGTGGGGTTCCATTCGATCCCCAAACCCCATATATACCAAGGACACCCACAACCCCACTTTTTTTGTTTCTCACTCTCTCTTTCCATATTTTTCCTTTTCCTTTTTTTTTTGTTTGTTTATTTTTTGTTTTTTTGAACAAAATGAGGTAAGGATTTTTCATTTCTCAATTAACCTTGTGGTTTCACCATGTACAATTTTACAATTCTAAGCAAGTTGTACGTCAACACCGATACAAAACAAAAGTTTACGCACTTCACTTCCCACAATCACCCCCTCTTGTAAAAAAATACAAAAAATACTAGGGTCTGACATGTGGTCTGATTGCTGACTCACAAAGTCACAATTTACCTCCAGCTTTAGGGGCCGGTCCAATGGGTTTTGTATTACTGTATTAGTCCAACAAAGATGGAATCACTTAAGTTTTTTAAAAAATAAATAAATATACACAAAATTCAGAAGCAAACTGTTGATATAATTATAAAGTTAGAAGATAAATGACATTAAGGCCATGTTTGGTAAATAATCAGCCTATCAGCCAATTTTGGCTTATTTGATCACTATTAGTTGGTAAATGATAAGCTTTTTGTAACTCCAAAGTGCTAAAATTCAAAAGGCTACTCAAAGCAGCCTTTTCAATTAGCTTTTTGAGAAAAAAAATTATACCAAACAGCTATCAGCTAACAGCTAATTTATCAAACAATTTCTTACAATCAGCCAATGTTATCAGCCAATGTTATCAACAAATCATACATTCTAACCCAAACAGCCAACCCAATCAGCTAACAGCCATTTACCAAACAGGGCCTAAAAAGTTTTGGCCAAAGTACTCGAAATCAAGTGTTTACTATTACCCTTAAGAAAGCCCAACTTTATTTTCATTTACTTGCTTCATAGTCAGTATCATGTTTCAGTGACAGAATGTTAGATTCGAATTTTCAAGTCACCGCTCAACAAAAGTCAGTTCGCAGTAAGAGTTAAACTCTGCAAACTTGATCGGCACCTAAACCGAACTGGGATTAGTTTGGTGGGGAATATGAGATGTTATACGCAAAAAGAATACGTACATAGGGCAGTATAGTGCCATTATTTCCCTCACCATCAATACGGTATTGCCGACCAGACCAGAGCAGTCCCATCTCATCCATCCATATATTTTGATCCCCTCATCATCATCTTTTTTGTAGACCTTCTCTCTCTCTCTATCTCTCTATACATACATCCCATGTTTATAATTACTATTATCTCTCCATCTTATTTAAATTTGCTTTGAATTGCCTTCTTTGTTACCCTACTACTGCTCCATGATTTCCCTTTTGTTTTTGTCATTGAGCTAGATAGCTCTCTCGCTAGCTATAATTTGCTGCACTGCAAAAGCTTCTTGATTTGGATAATAATCTTTCCTTCTTTTTTTTTTTTTTCTTTCCATATATTTAGCCAAAGCACCACCACTGCCACCACCACCACCACCATCTCTGATCCGCAATTCCCGGAACCTATATATATAAACAAAAAAAATTTATAAAAAAAGAAAAAGAATACAAGATACCCTTCAATGGATATCTCTTCTTCCTCTTCCTCTTCCACTACCTCGGAACGGGTTTGTTACGTTCACTGCAATTTCTGCACTACCATTCTTGCGGTATACTTCTTTAATTCTCCATTTGTTTCTAGCTATATAAGTAGTAGGTGTTATATAAGAGAATTCACCATGCATATATATATATATATATATATATATATATACTAATGATTTTCAAGCCATATATCTCACAAGACTCTTTTAGATTTCTTCTTCTTCTTCTTCTTTTGTTGTGTTCTTGTGAATGATATGAGGAATTAAGGTAGAAATTGTCCTCAAGAAAAAGGGTTTTCTTGGAACCACAAATTTTCTGCAAATTTGTTTGCAAGTGAAGTACTTGTTCATCAATCATAACGTGTTAATTTAGTGGAATTTAAGGGAGAGGGATTTAGTTGTGTGATATTTTATGCAAATTCTGTGAATTGTTGGTTTCTTCTTATTCCAAATTAAAAAGTTTCAAAATTGTGAGAAAAAGGGTAAAGGGTGGTTTGGTTTAATTTCCAGTTGTGAGAAATTTCTTGTTTTGAAGCTACAATTCAAAAACCTCAATGTGAAAGGGAAAGGAAATCGCGATCCAATAACATGAAAACCCCATGAATCTATAGGATAAGTGCATCAAATTGATTAGTCATACAAGAACTATCTTGAAGCTAGGGATATATATGATATGATAGAGAACTCATCAATATATATATACATATATATCTTGGTTTGGATTTATAATTAGGGTTTTATTTTTATTTTTTATTTTTTTTTTATAAAAAAAAAAAAGTTTCTCGATCAAATGGGTGGAGAAGAGGAGTTTCTTTGTGCACTCCAGGTTGATGAATAGCAGTATATCTTAAGGGAATTAATTTAATTTTAATTAATTGATTACACTTGTTTTATTAAATATATATTTGGTTTTAATTTTCTGATATTTTCGTACTTGTGATCTGCAAGTGATTACAAATAATTGTACTAATTTTGATGCCACTATGGTGGTGGGTATTTCTGTTCACACCATTAAATTCCATTTCTACATCCAACACCCATTCCAAATCTTTCATCTGTATAACAAAGTCTGCGTCTTTCTTTCTTTCTTTCATTTAAAATGGCCTGGGTATACTTTATTTTACATATAATTAATAAAATAGTTTTGTTTCTTGAAAAAAGGGAATGATGGGGGGTGATCTTAGAGACTGCACAAAGAAAGAGGTGGATTTTAAATTTAGTTTAGAAATAATATAATTAATTATATTATATTGTAAATTAAATAGAGTCAAGTCATTTTCGTTTGGCCCTTTCCCCTATCTCTTTCTGTGTGAGTAGTGACTGAATCACTGAACAAGTTTCAGAGATAAAGTCAGGAGATGAGCACCGAAAGGGTTGGGGGGGAAAAACAGAAAAGGAAAGAGAGATATTGGTGAGATTAGAGGCTCAACTCTCTTTAAAGGGAACTCCTTATTATAGCTACTTGTTTTCTTTTCTTTTCATTTCTTATGTGCACCAATAATCCTTGGTTTTTGCACTCACAAAAAACTCAATTTTGAAAACAAAATGCATACCATTAATACATTTATATATATACCTCATAATAATATATATAATGCCTGCCTTTCTTCAATTCTTCCTTCTCTGATCTGTATAGAGTACATTCAGTGAATGTTTTTTTTTTTTCTCTCAAGGTTACTAAACCTGGTAATTTAATGATTTACTGTGCTTTTCTCTTTCTCTTTTTGGTTGTTGCAGTAATTTGTATATCCATTTCTAAATCTAACATCATATATATCTTTTTTGAAACTTTAATTTCCCCTTCACAAGATGCTGATCAGGTATAGTAGTAGATAGATCCATCAGTGCTGCTTGTGTTTTTGCAATTAATAATATAATGTGGATATTTTTATTTATTTTCTCCTTTTTAAACCTTTATTTTCTATTTTTCCCAATTCTTGCCATATATATACATCTGTATTTGTTTGTTTGTTTGTTTCCAGAATGAATAAATTTGAGAAAATTGAGGTTTGAGAATGGAGGGTGACATAAAGTAATGTTTGATTTGTTGCATATTTTTTTAGGTTAGTGTTCCATGCAGCAGCATGTTCACCATTGTGACAGTCAGATGTGGGCACTGTGCTAATTTGCTTTCTGTTAATATGGGATCTTTGCTTCAGCCTCTCCCTCTTCAAGATATCCAGGTAATTTACACTGTTTCACATTATATAAAATCAAGAAATTAACTTTTTTTTTTTTTCTCTCTTTAACAACTTTTTGAATGGTCAAAAAATAAAAAAGGACATGCATATAAACATTAAAGTTTCTGACTACATTATACATACACACATACATACATATATGTGTTTGTGTGTATTATAGATCTGCAGTAAATATCATAATGTGAATTATCTGCACCAATGCTTTTACTGGCCTCCCCTTTCATGTCCTTTTATATATGTTACAAAATTTCTTATTAATTTCTTTTTATGAATTTGCAAACTTCAATTTATTATCTTCATAAATAGTTGCCTGCCTAGCTAGCTAGCTTCTGTTTTATGGAAATTATAATAAAACTTCTTTAACATGTATATAATGCTATAGTAAGTAAAAACAAATCGGCCTAATTTCTATTGTGTAATGAACATTATGTTAAATAATATAAGAACTTTAGCCTATTGTAAATTAAAGGTATTCTATGATTATATGAAAATTCATACATGTTAAAGAAAAAAATAGTATATAATAAATTCTTAGATATTTTTACAGCATGCATCACATACTACAAATATCCATATGGGTATGCTGTTGAGGCCATGTTATAACACTGATTTTATAGTTAAGATAATTGAAGCATGTGCACTATCTCAACTAAAGGCTAAGCTGATAGCTAGACTATGTTTATATGTTACATGCTCAACAAAGGTTAAGAAAAAACATTTTATTTTGTGTGTTTGTTCTGTGCAATAATGTTTTGTTGTGAGAATGAGCAGAAGCAACAGTCCAACAATGAAGATGGCAGCGGATCATCGTCTTCTTCAAAATGCAACAAATTTGCTCCCTTTGACTCTCCTGAACATGAACAGCAACCCCGACTCGCCCCCATTCGCCGTTAGCTCTCTCTACTTTACTTTAAGCATACATTTAACGACATTCCTAATTAAATCGATAACCGTTACACAAATTTTTGTGTCTATTAGTAACTTGATTTTGAGTTAATTTAAAAGTAATAGTGGTGTTGATATTTTTTATAAATAAATATAATATATAGTGAGTGGGATCAAAAGATGTTTATAGGTAGGAGTCCAAAAATTGGATGGGTTATGTTCATTTATGATCTGATATGATTGTGACAAATCATAAAATCCAAGTCAGCAATAAGAGCAGGCCATGCCATTTTCTTGAGAGATGGAGGGAGGGGATTTCTGTACGTGTTCCATTTCTTCATCATCATATCGCTTTACTCTACCGTCTAGCCTCTATTATTGGCATAAATAGGTTTGGATGAGAACTACTGCCATATCCACACTACCCCAAAACCTCCAAAATATATATATATATATATATATATATATATATATATATATATATATATATATATATATATATATATATACATACTACGAATTGATTGGACGACTTGCAATTTATCTCCTCCAGTGCTCTTATATACTACGAATTGATTGAACGACTTGCAATTTATCTCCTCCAGTACTCTTTGGACAGGGGTTCTGGGAGCTTAGGATTAATGTAGTATAAGCTGGGAAACCTAATCTATTAAAAAAAAAAATACTAGAAAGAATATTCTTTTGCTATGAAAGTATTGGTTCGAAAAAAAAAATGAAAATGGGTAGCATTTAAAATTTATTTCCAATCTCTTGAACATTTAATATATATAACAGTTCAATTATCAGTTTAGTCTTTTAATTGAAACAATACATATTTTCAATTTGATATTAGATGGCAGAGTTGGAGTTTTGAGTTGAATACTAAAATACATACATGCATGCTTGTATTTTATGAATTATAAATTTAACATTGCATGTGATACTTTAAGTAATCATATCATATATGCGCCTTCCAAAGATTTATAAAGCATGCTGAGTACATGTAAAAAAGTAACATTTAGAGTATTAAGATACACATAATAATTTTGTATAAACGAGCTGAGTGTACAAGTTTTATGTAGTATTTTTATATATACTAACATATATAAATAATAATGATGAATATAATATAATGCAGCACCAGAGAAAAGACAACGAGTCCCTTCTGCGTACAACCGATTCATCAAGTAAGTTGGAAAATTATTTAATTGAATTTGTTTATGTAATTAATAATATTATGTTCTAGCTAAGGTTGCTGACAAGCTAGCACACTATTTGGGTACGTTGTTTTTAATTAATGTATGTACTAAGTAGGCATGTATGCATTCATCACATGTTGTGTTTGTATGTGTTGTGTAGGGAGGAGATCCAAAGGATAAAAGCTAGCAATCCAGATATTAGCCACCGGGAAGCTTTCAGTACTGCTGCCAAAAATGTAAGTCATATACTTATCACAACAAATATATATATATATATATATATATATATATGTGTGTGTGTGTGTGTGTGTGTGTGTGTGTGTGTGTGTGTGAATATTTGGCTTACTAATAAATTCATTTTTTTTTAGTATAATCTGATCGAATACTGCATAAGACTAAAACCCACCTATTTGCCAGGTTATAAGTTGGGTTAGCATAATATATTCATATTTAAATATCTATCTAGTTGACTAGCCGAGAAATCTTTAGACCAAAGCAGCAAATGATATACAAAAATCCACCCTTTGTTCTCAACTCTACTATGTGTTAAAAATCTAGAGATTTTCGTAGTATCAATCTACACCGTAAAAATTCCTAAAAATCTAATGTATTTTCATGATTTAAAAACTAGGGCTTCATTTAATTGAGCAAAGGTACAATGATGCTACTTCTAATATAATGTATTTTCTCCATTTCTATAATTCAGAATTATATCAAAACATTACTATTATATTTACAACTTTTAGAGTATTCTAATAATTGAAGCATATGTTCCATCTCAACTAACTATATATAACGTAATTAAGTTAGTCTGGAATAATTTGTAATGAATTTTTATTATATTCTCACATTTTATTGGCGTTCCAATAAATACGATGAAAAAAATCACTTCCTACCATTGTATTTTTGGTACTCATAAATTTTATATTAAAACATATTGCAAGCTACAAAGAATCTTTAGGTATATAAGCATTTGATTTCTTTTTAAAGATTTCTTGGGGCAACAATAGAACAGACAACACATGAAACAAATGATACTATTTGGTATCAGGGTGGGTATTACATTTTGGTTTCTTGGATGCCCTCAAAGAATATATATATATATTCTTTCTCTCTCTCTCTAGACCTCTCATTTAATTTGTTGAATAACTAAAAATGATTTGCAGTGGGCACATTTTCCACACATCCATTTTGGACTAAACCTGGATAACAACAAGCAAGCCAAGATAGATCATGCTGTTGGAGGGGAAGGGCCTCAGAAATCACTTGGTCTCTACTAAGAAATAACATTTCAAGATCTACCAGCTTACTTATATATATATATATATATCTATATATATGGATAAATAAAAGGCTCATATATATCTATATATATTTCATATATATTTCTCTATATATATATATGTATGTGAAGATTTGAATTGAGTGTCCAAACTGAAGAGTTTGGAATTCCCATATGTCCATCTGATGAGGTATATCTTTGTAATATCTATCCCTTTTCGCAGTGTGATGATGATTTTATCCCTTCCTCAGAGGGCCTTGGATTCATTATTAATTTGTGATTTCCATCTTATATATAT

Note: the sequences marked in grey are exons.
