# Supplementary figures and images for: Resequencing of sweetpotato germplasm resources reveals key loci associated with multiple agronomic traits
Source: Hortic Res. 2022 Oct 19;10(1):uhac234. doi: 10.1093/hr/uhac234 (PMC9832839; doi:10.1093/hr/uhac234)

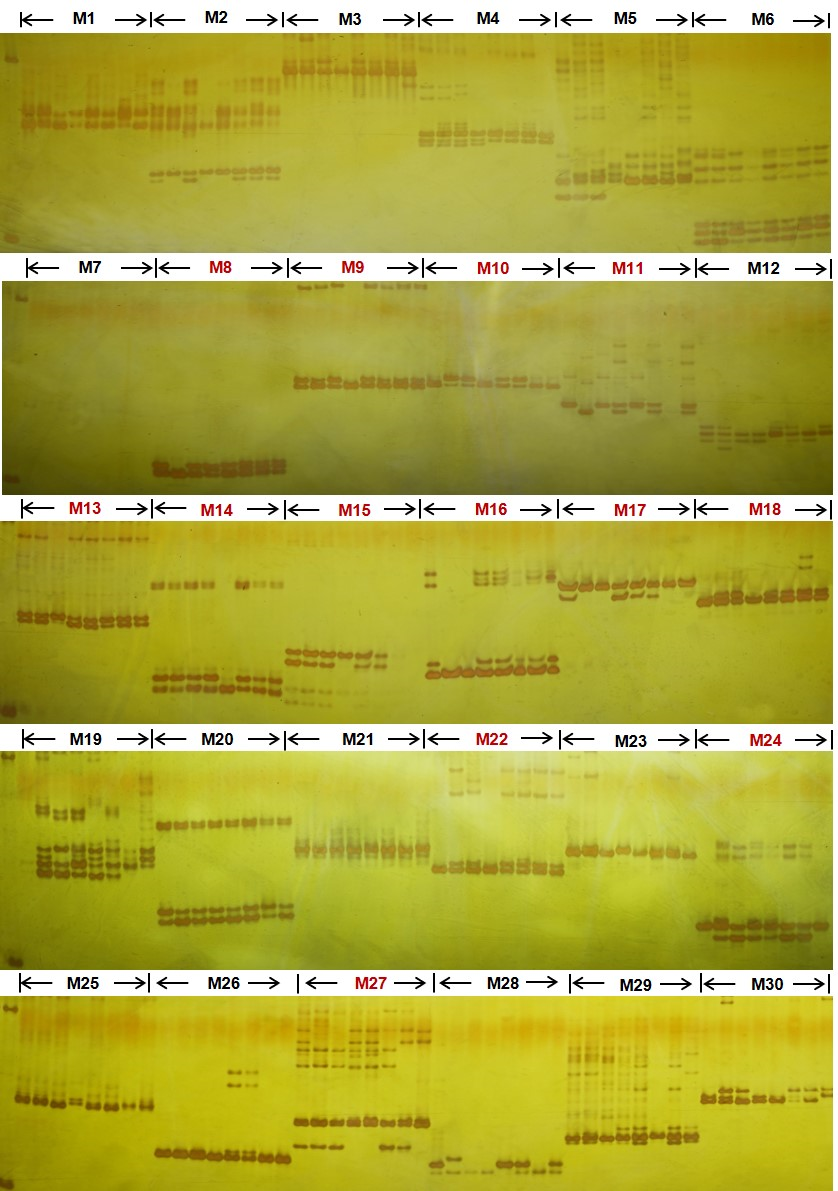

Supplement: Web_Material_uhac234 [file web_material_uhac234.zip › Fig.S1.tif]

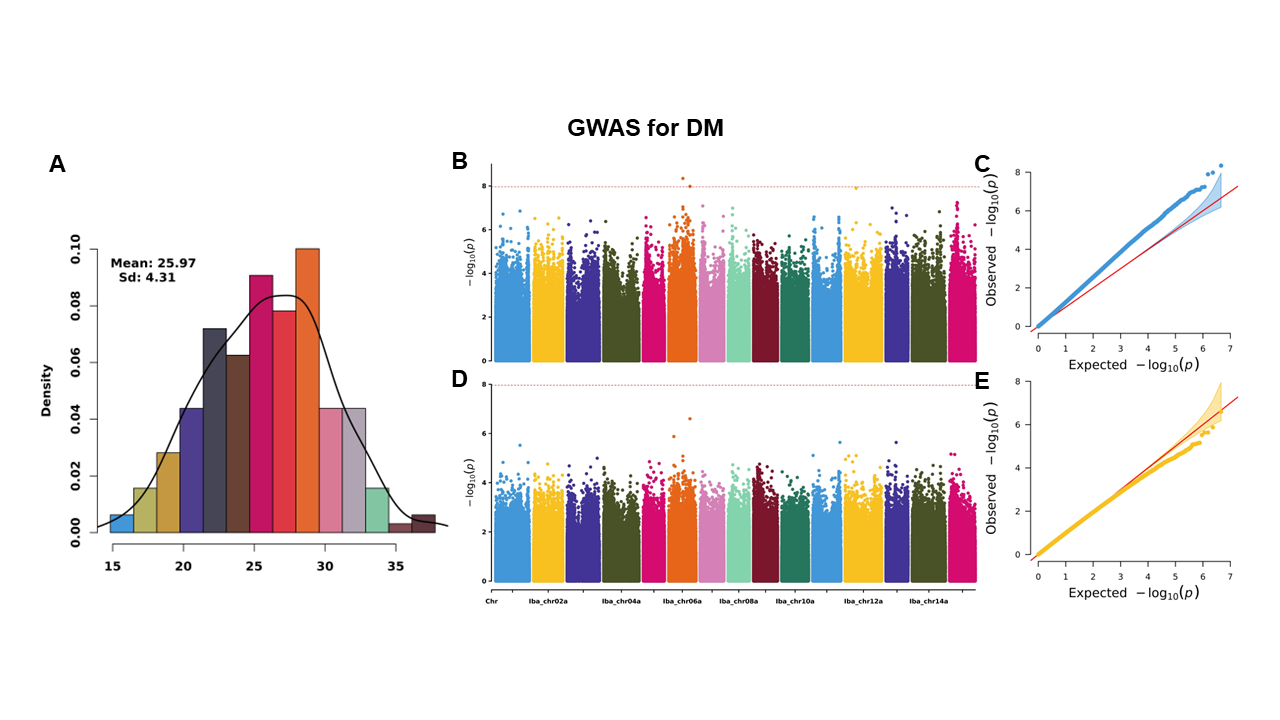

Supplement: Web_Material_uhac234 [file web_material_uhac234.zip › Fig.S10.TIF]

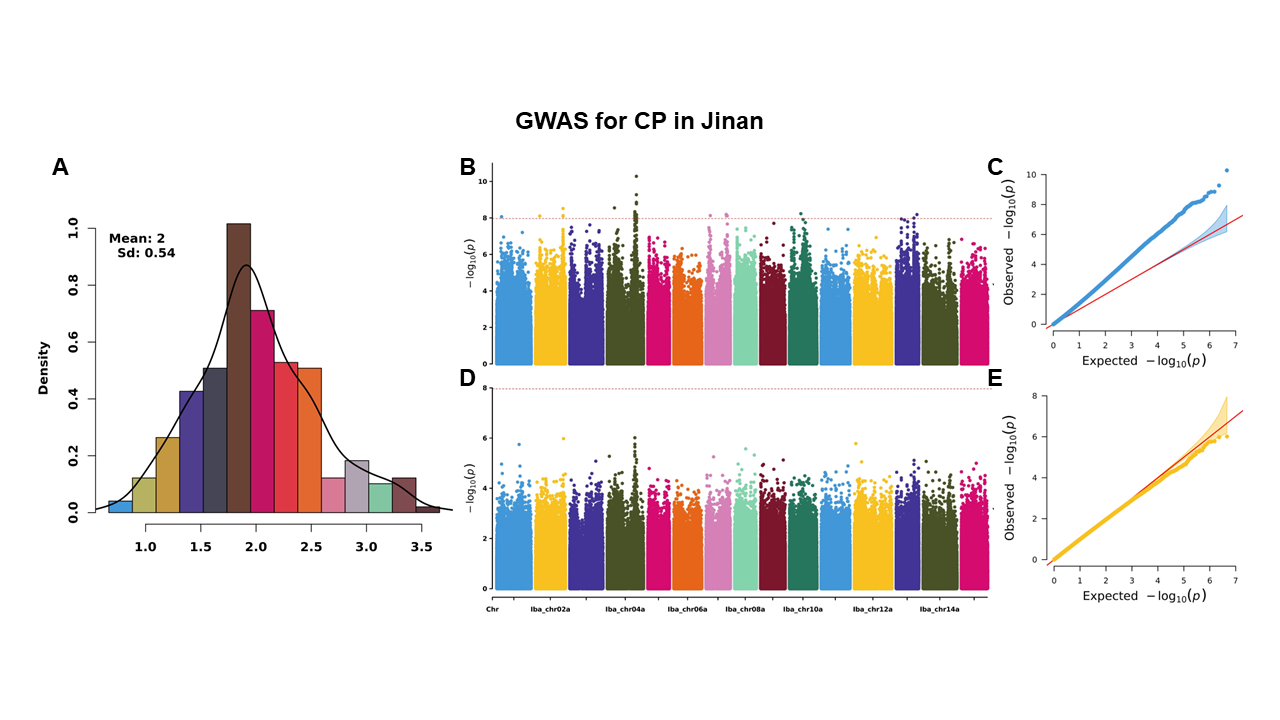

Supplement: Web_Material_uhac234 [file web_material_uhac234.zip › Fig.S11.TIF]

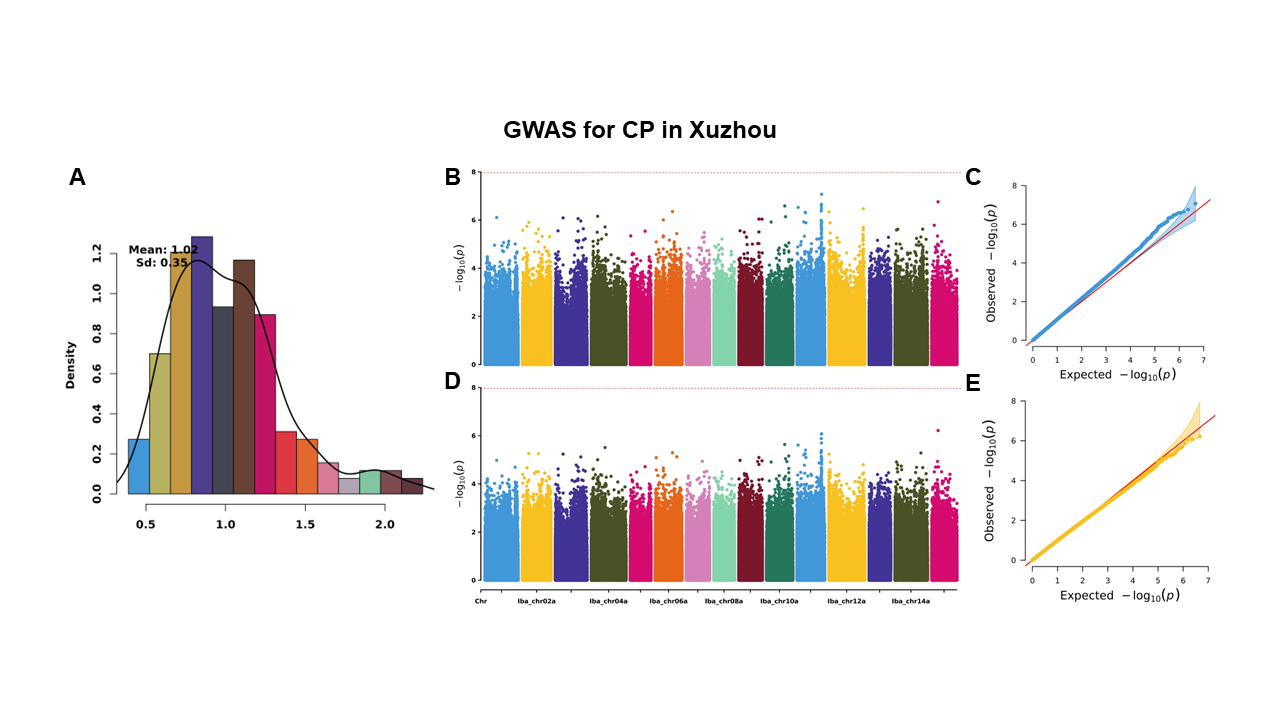

Supplement: Web_Material_uhac234 [file web_material_uhac234.zip › Fig.S12.TIF]

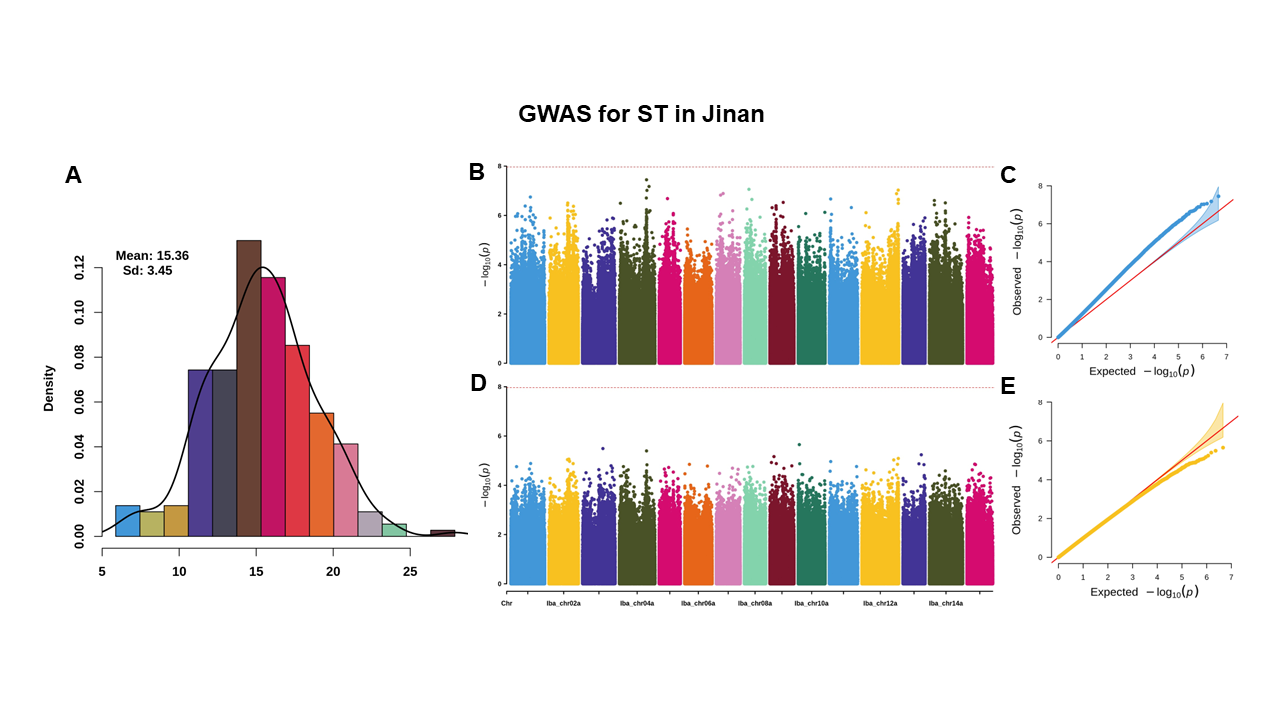

Supplement: Web_Material_uhac234 [file web_material_uhac234.zip › Fig.S13.TIF]

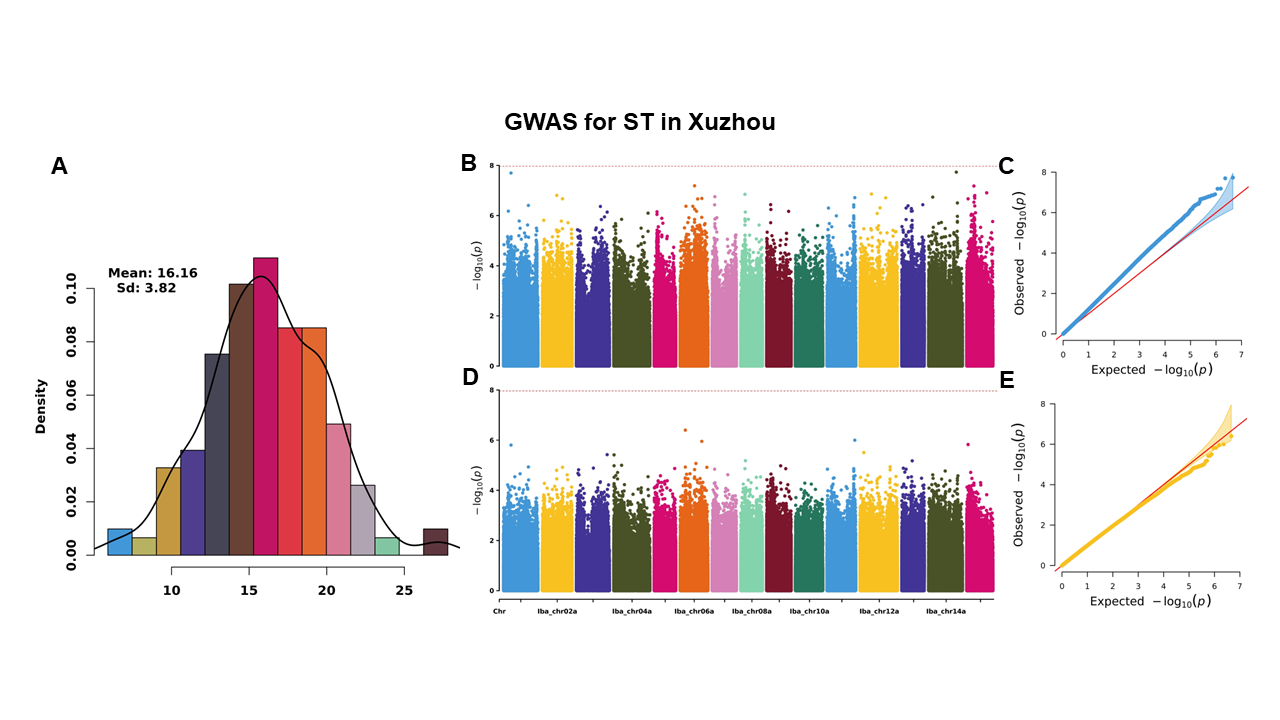

Supplement: Web_Material_uhac234 [file web_material_uhac234.zip › Fig.S14.TIF]

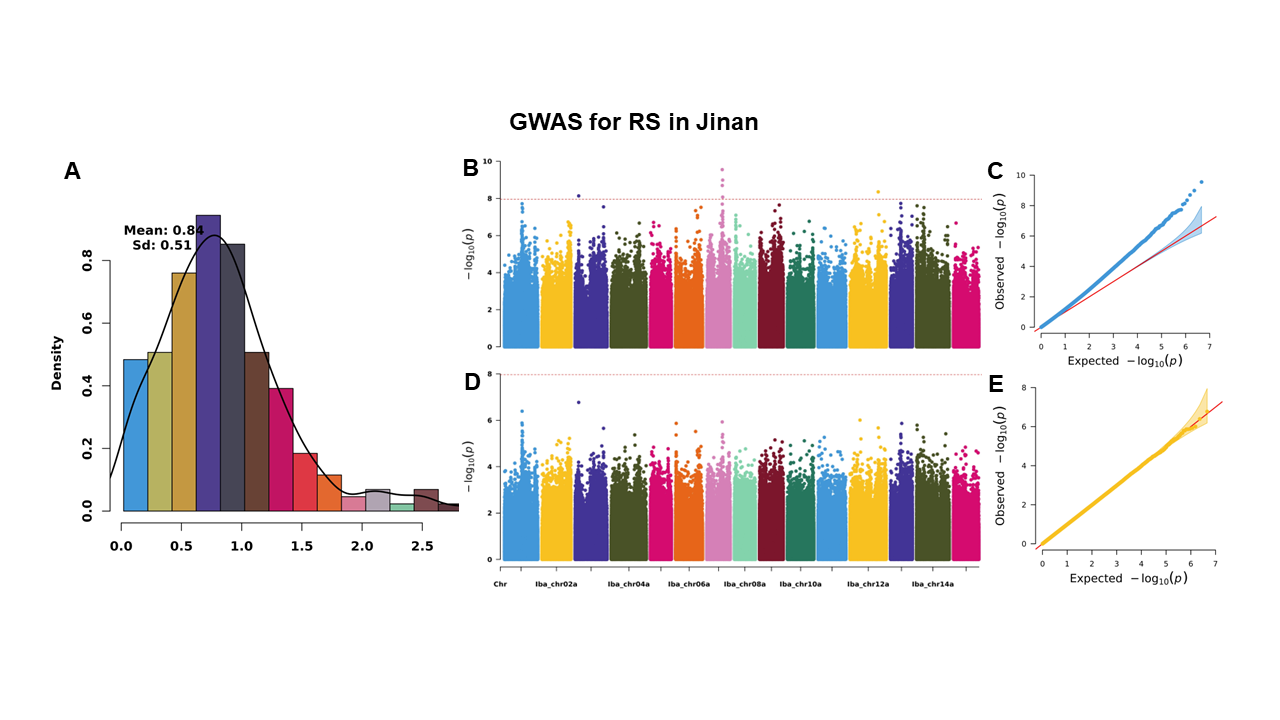

Supplement: Web_Material_uhac234 [file web_material_uhac234.zip › Fig.S15.TIF]

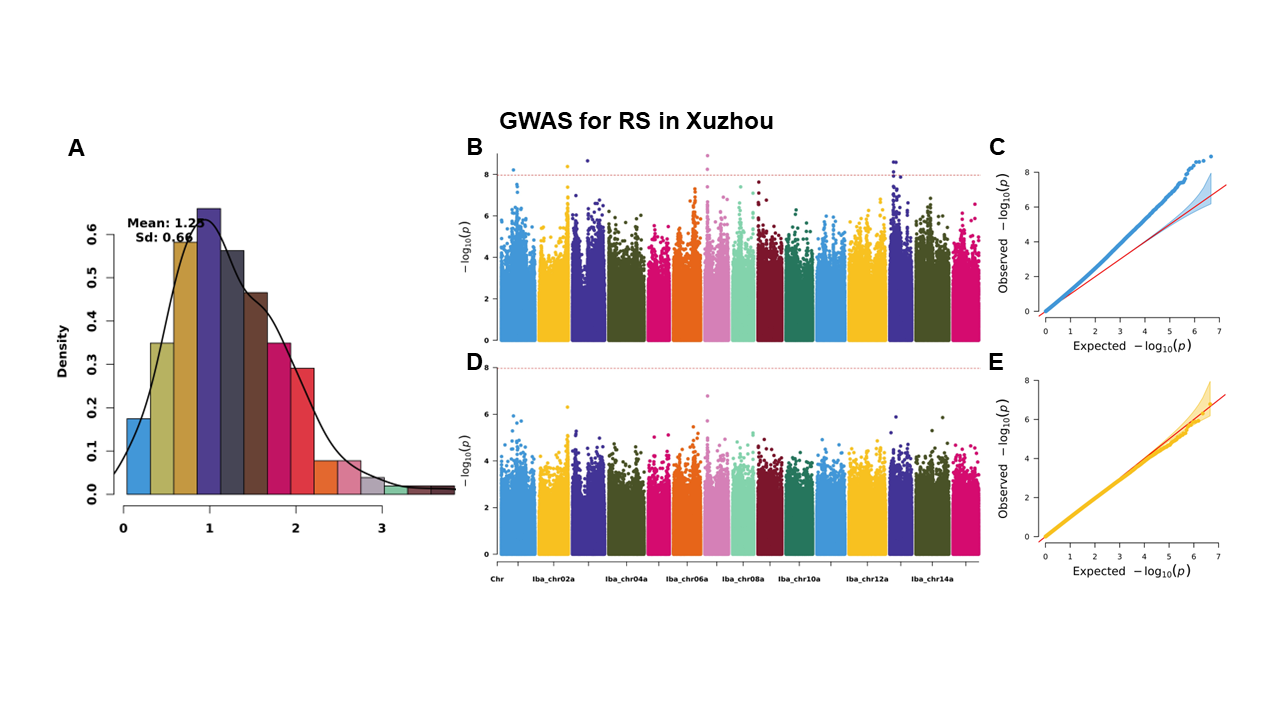

Supplement: Web_Material_uhac234 [file web_material_uhac234.zip › Fig.S16.TIF]

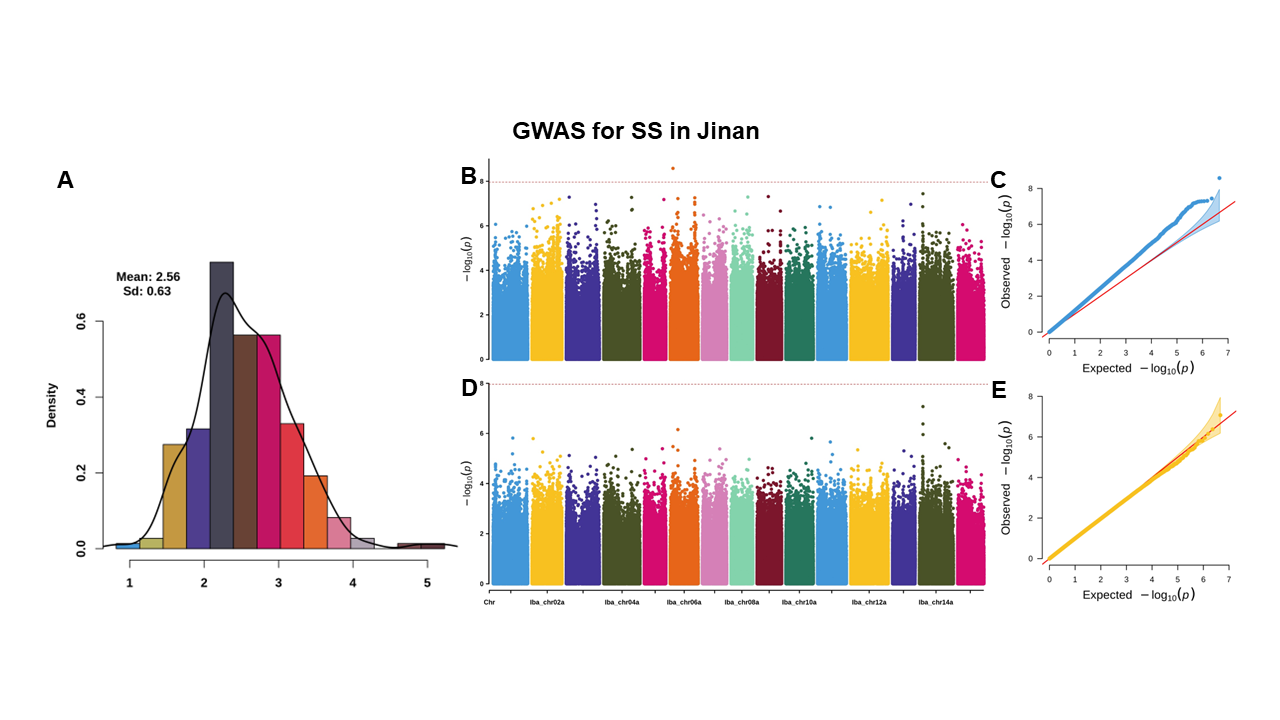

Supplement: Web_Material_uhac234 [file web_material_uhac234.zip › Fig.S17.TIF]

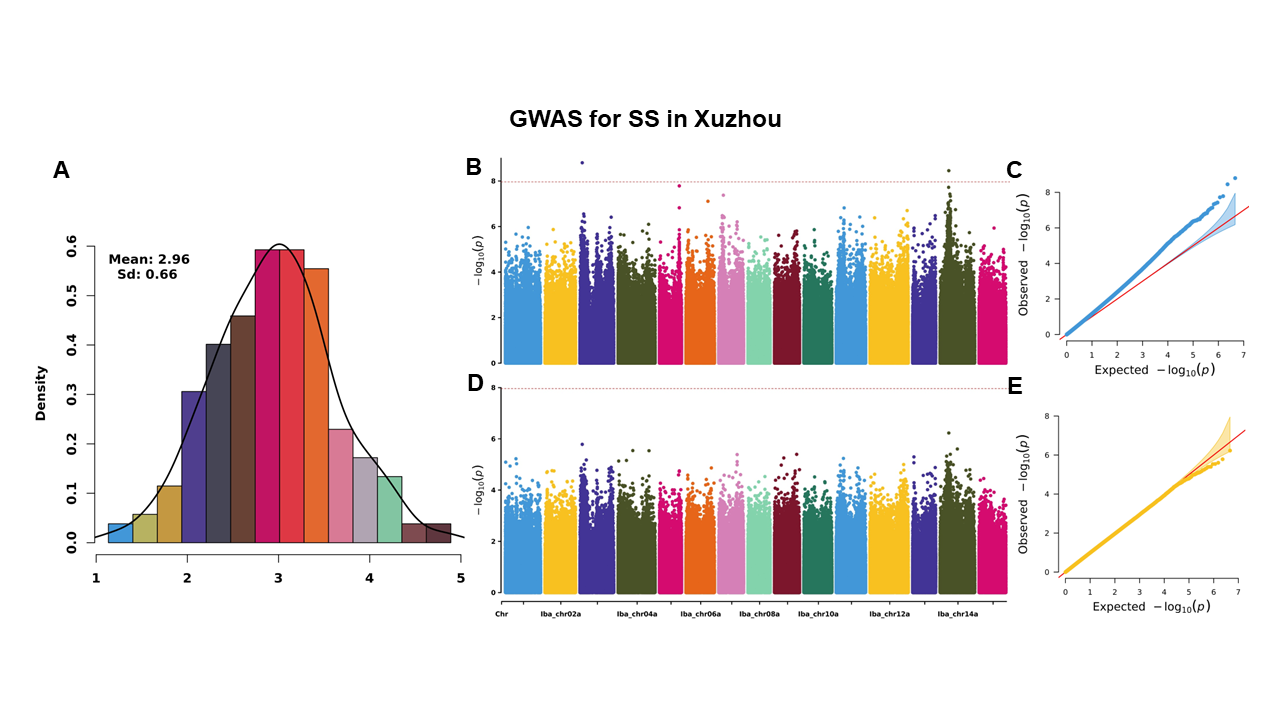

Supplement: Web_Material_uhac234 [file web_material_uhac234.zip › Fig.S18.TIF]

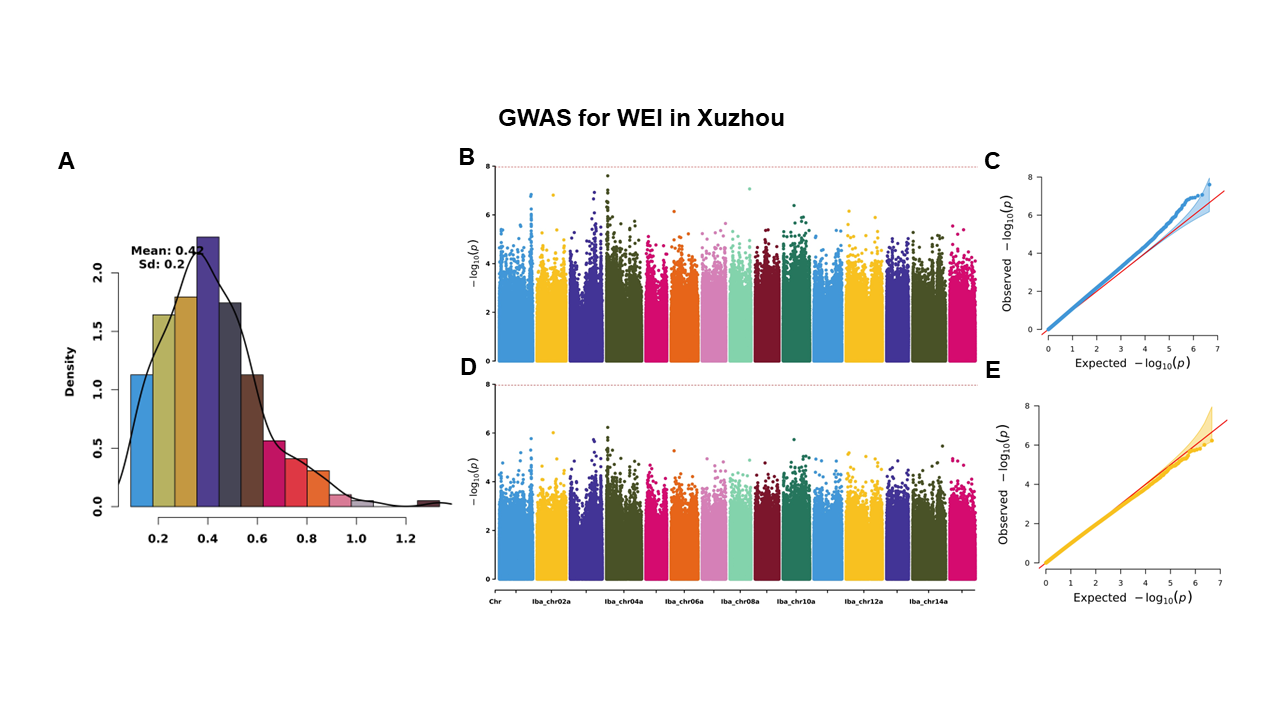

Supplement: Web_Material_uhac234 [file web_material_uhac234.zip › Fig.S19.TIF]

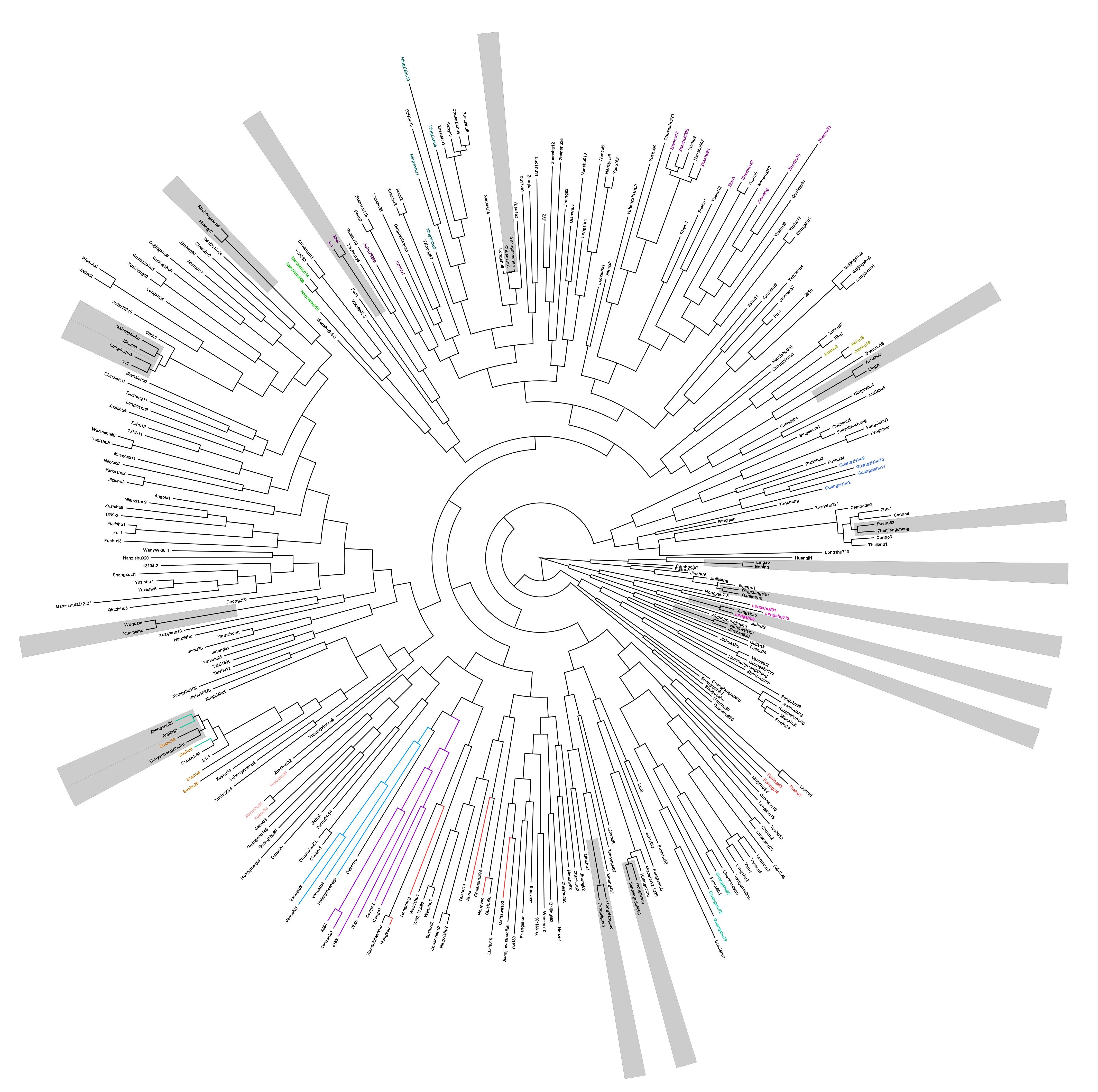

Supplement: Web_Material_uhac234 [file web_material_uhac234.zip › Fig.S2.tif]

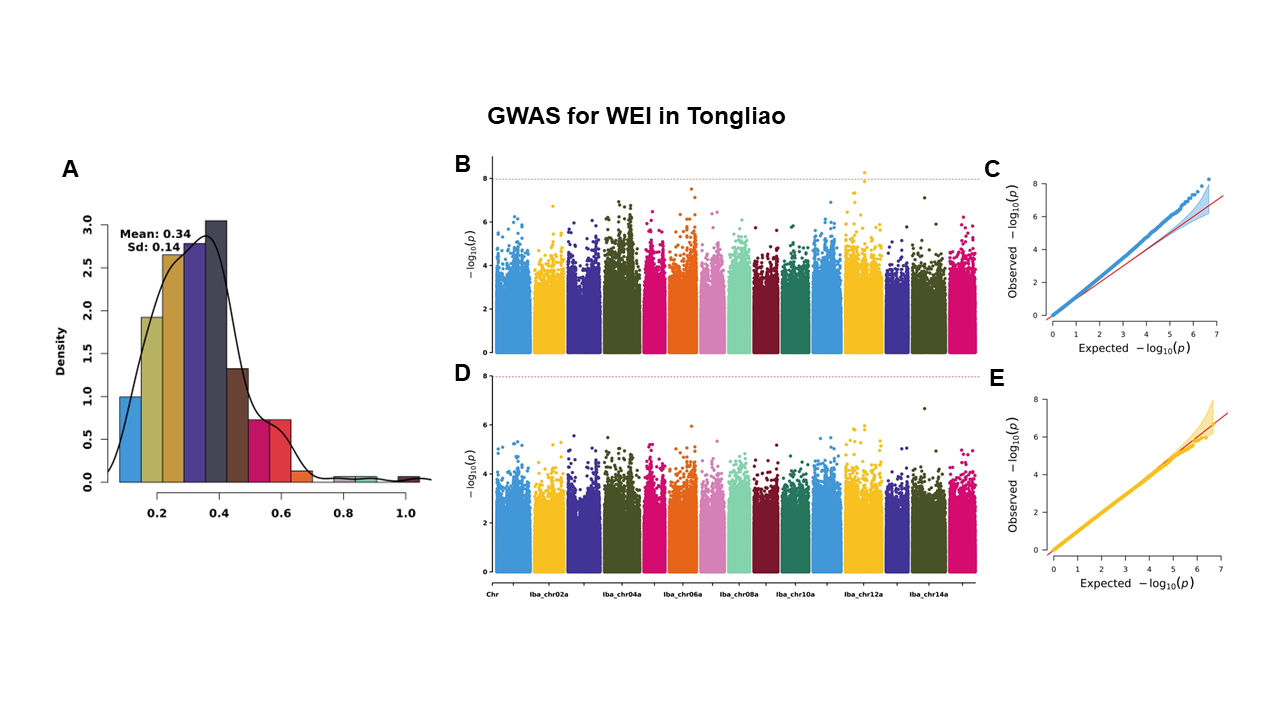

Supplement: Web_Material_uhac234 [file web_material_uhac234.zip › Fig.S20.TIF]

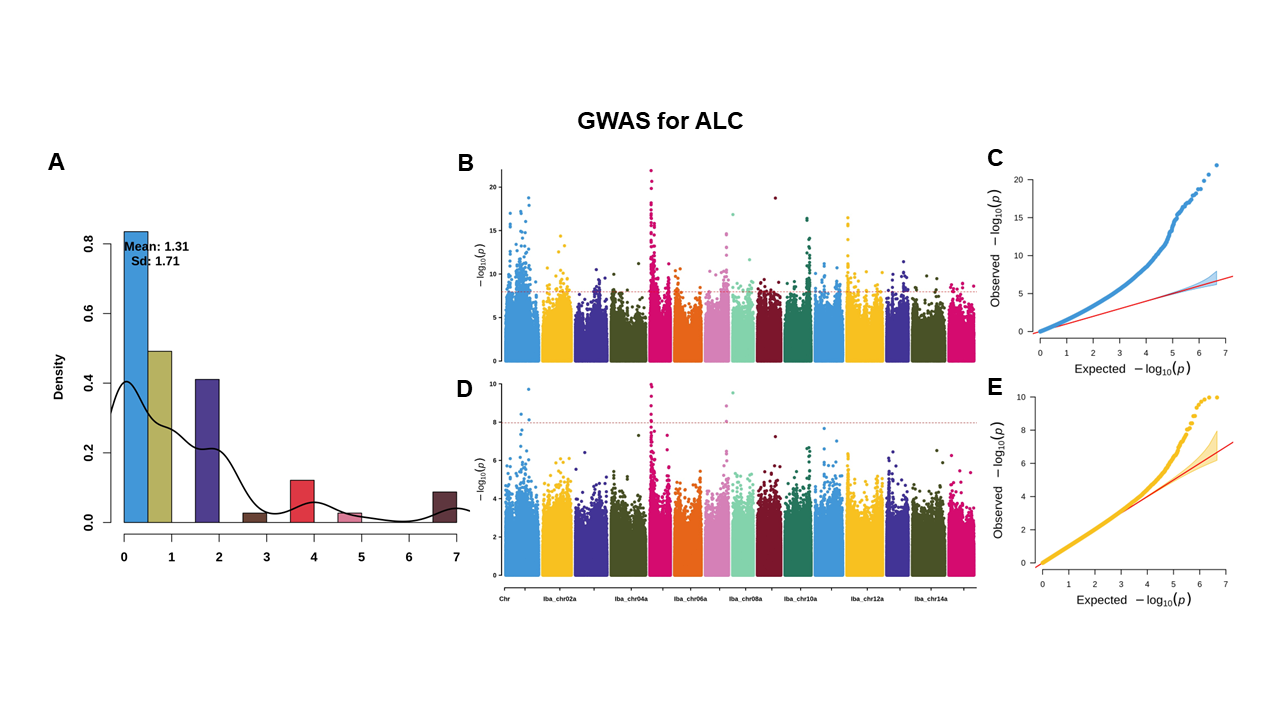

Supplement: Web_Material_uhac234 [file web_material_uhac234.zip › Fig.S21.TIF]

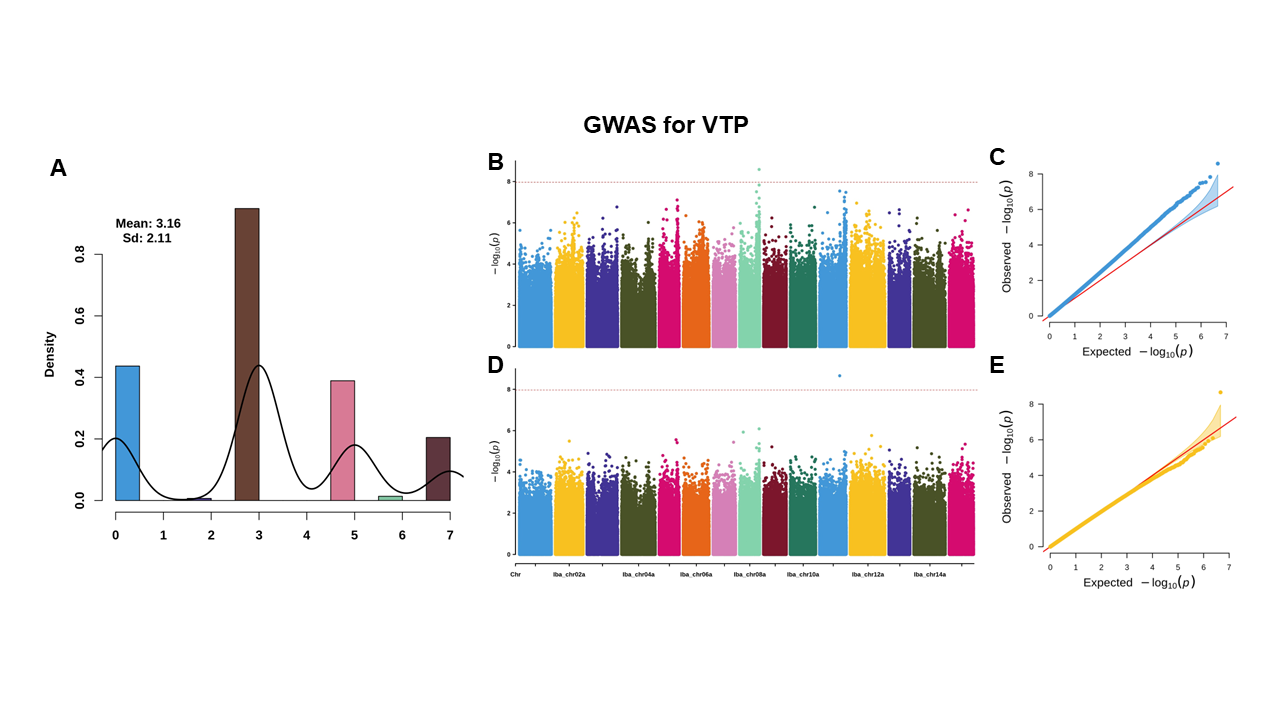

Supplement: Web_Material_uhac234 [file web_material_uhac234.zip › Fig.S22.TIF]

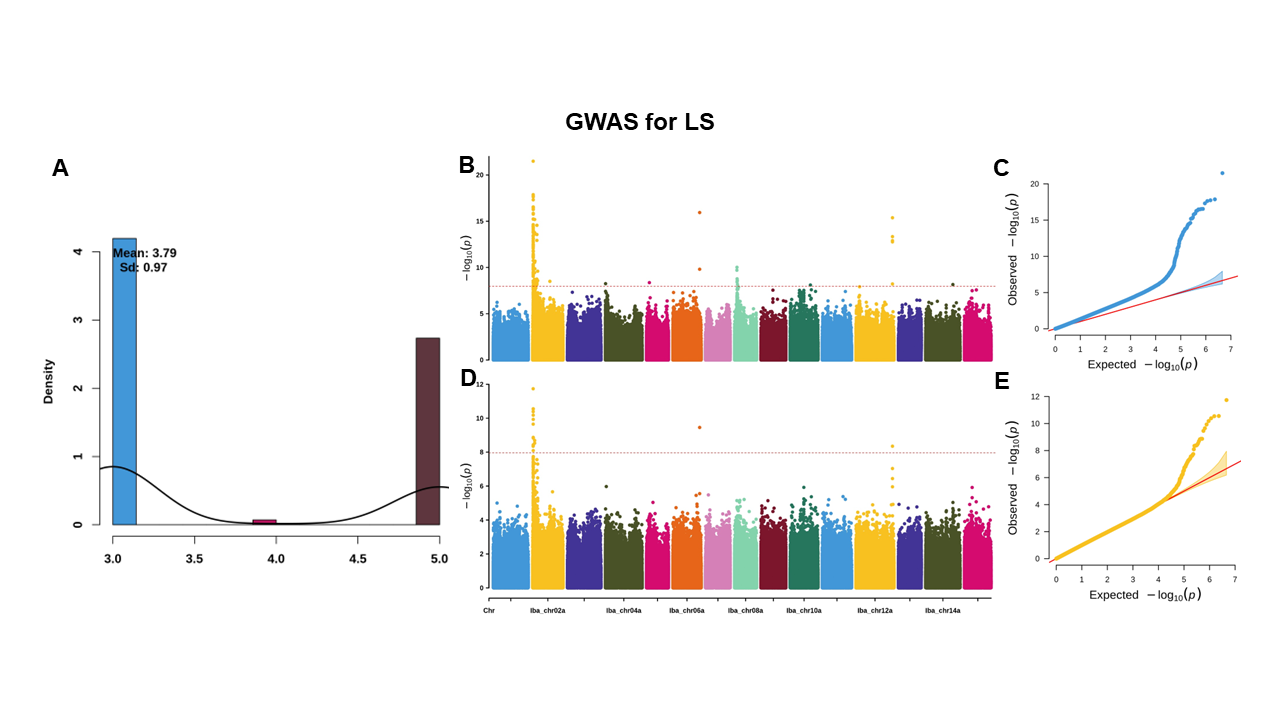

Supplement: Web_Material_uhac234 [file web_material_uhac234.zip › Fig.S23.TIF]

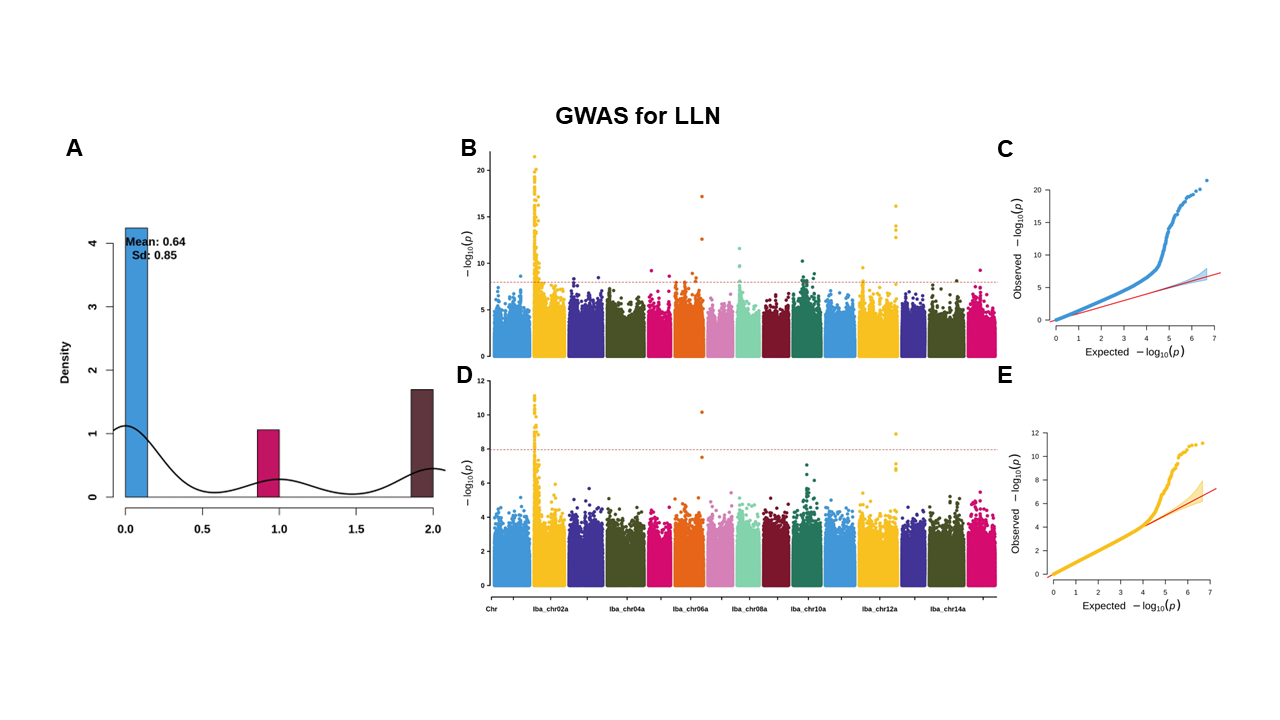

Supplement: Web_Material_uhac234 [file web_material_uhac234.zip › Fig.S24.TIF]

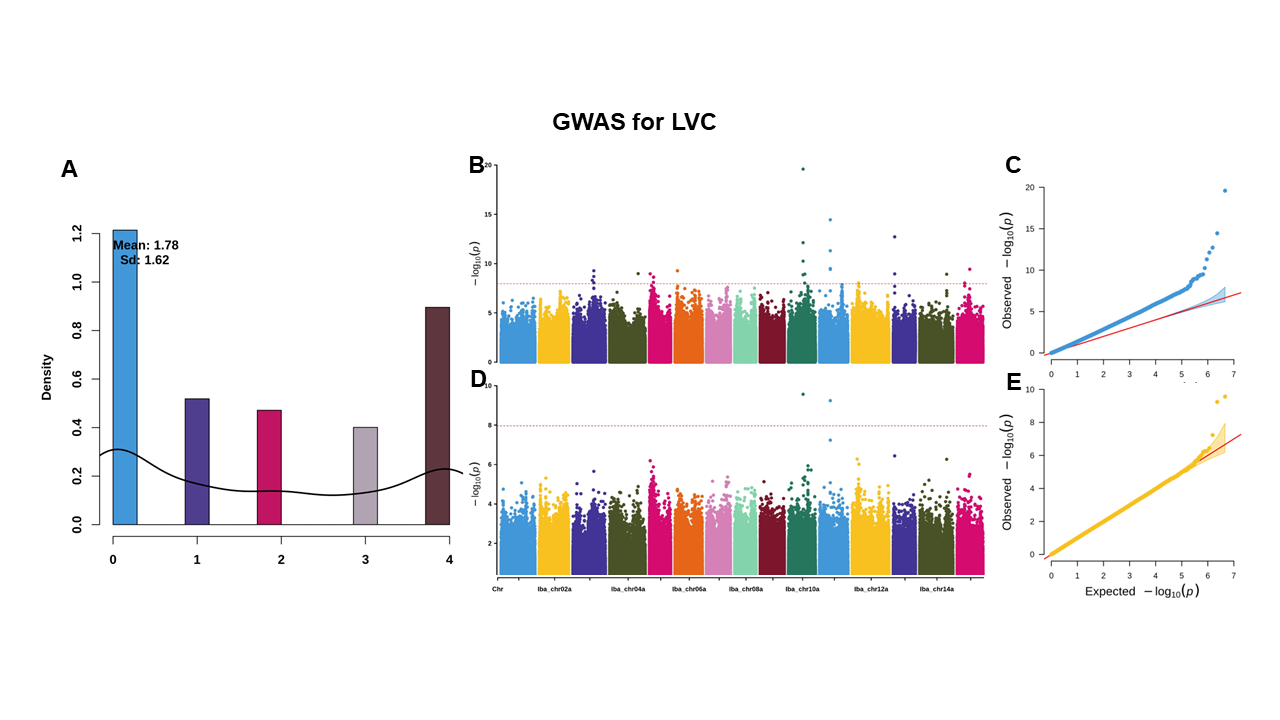

Supplement: Web_Material_uhac234 [file web_material_uhac234.zip › Fig.S25.TIF]

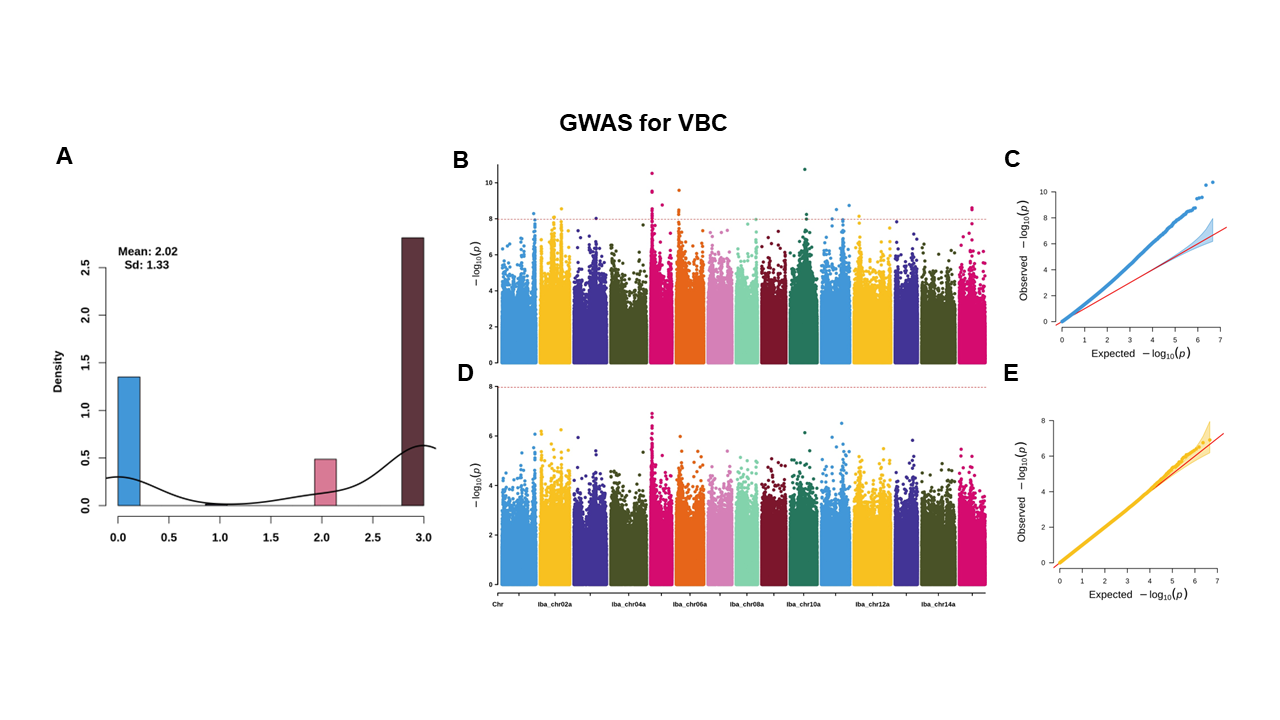

Supplement: Web_Material_uhac234 [file web_material_uhac234.zip › Fig.S26.TIF]

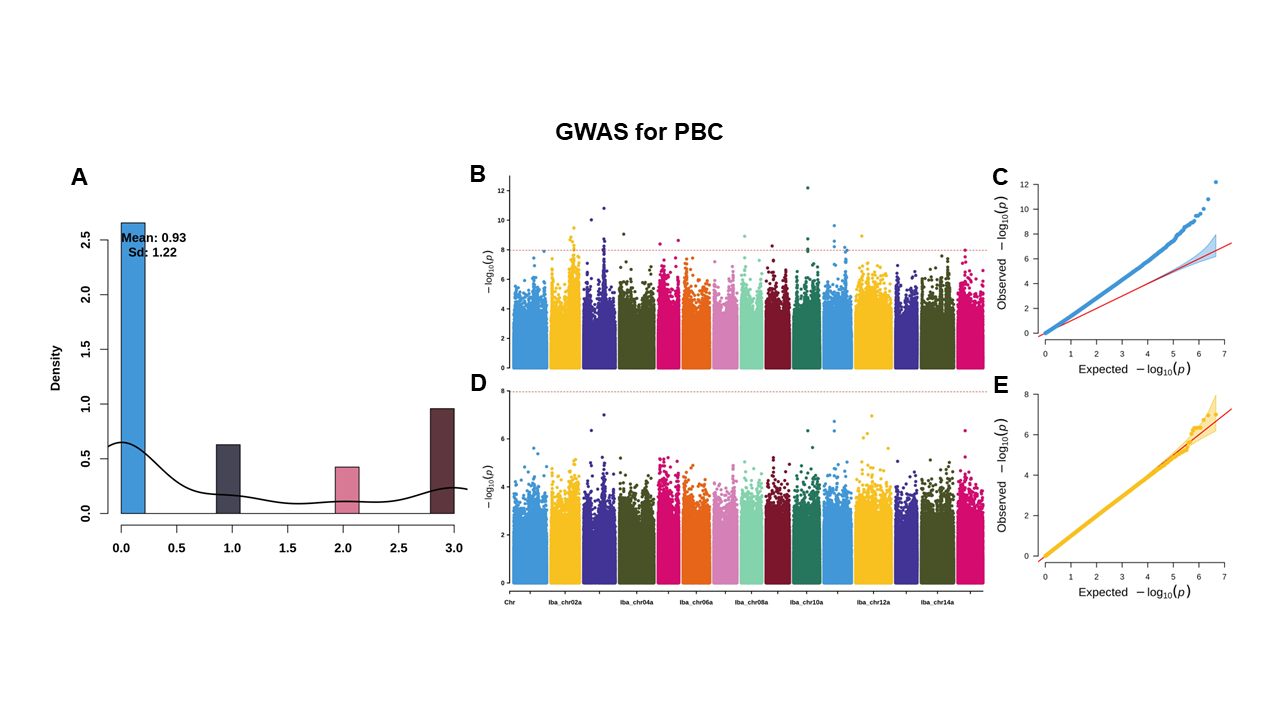

Supplement: Web_Material_uhac234 [file web_material_uhac234.zip › Fig.S27.TIF]

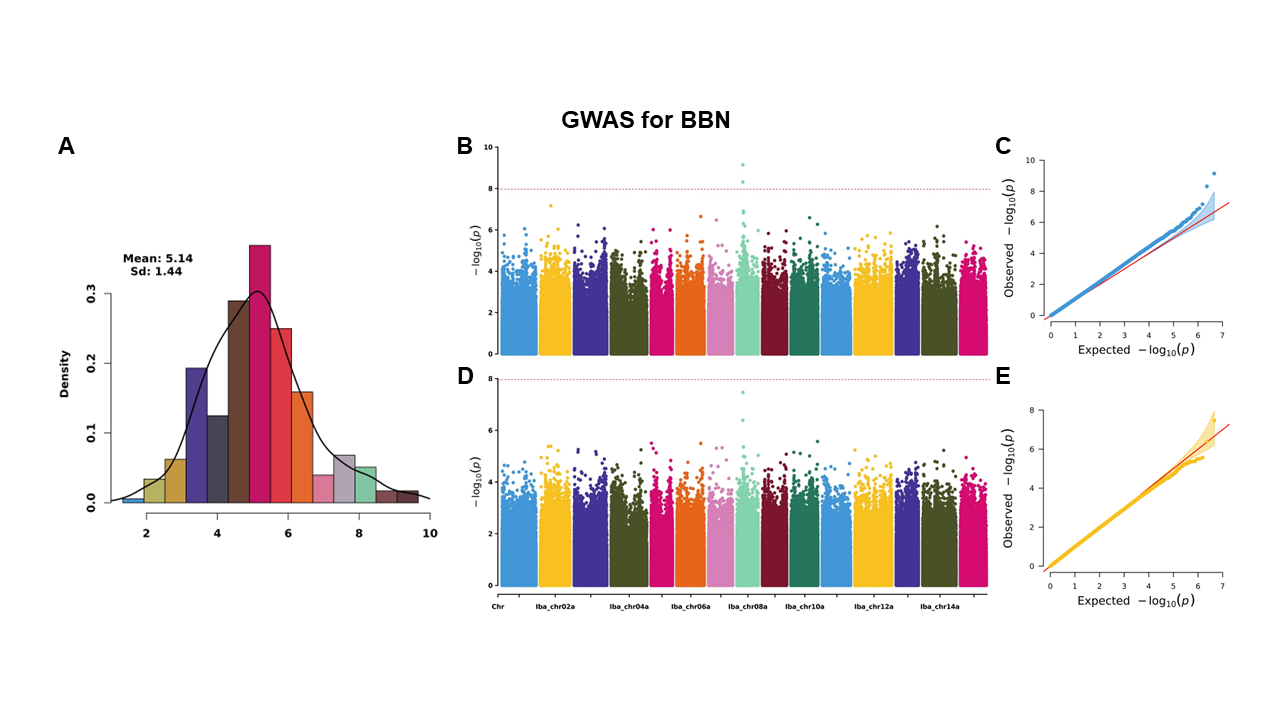

Supplement: Web_Material_uhac234 [file web_material_uhac234.zip › Fig.S28.TIF]

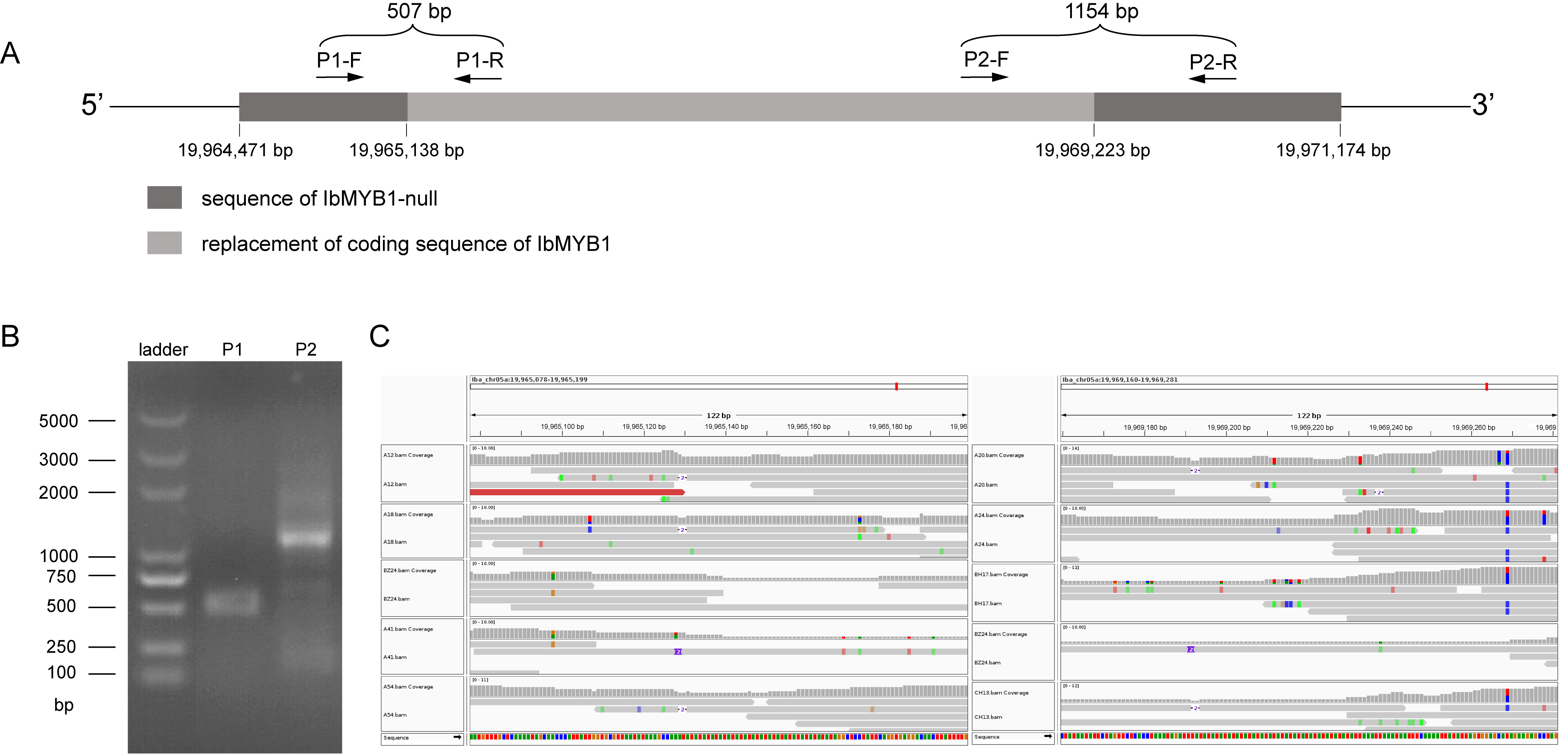

Supplement: Web_Material_uhac234 [file web_material_uhac234.zip › Fig.S29.tif]

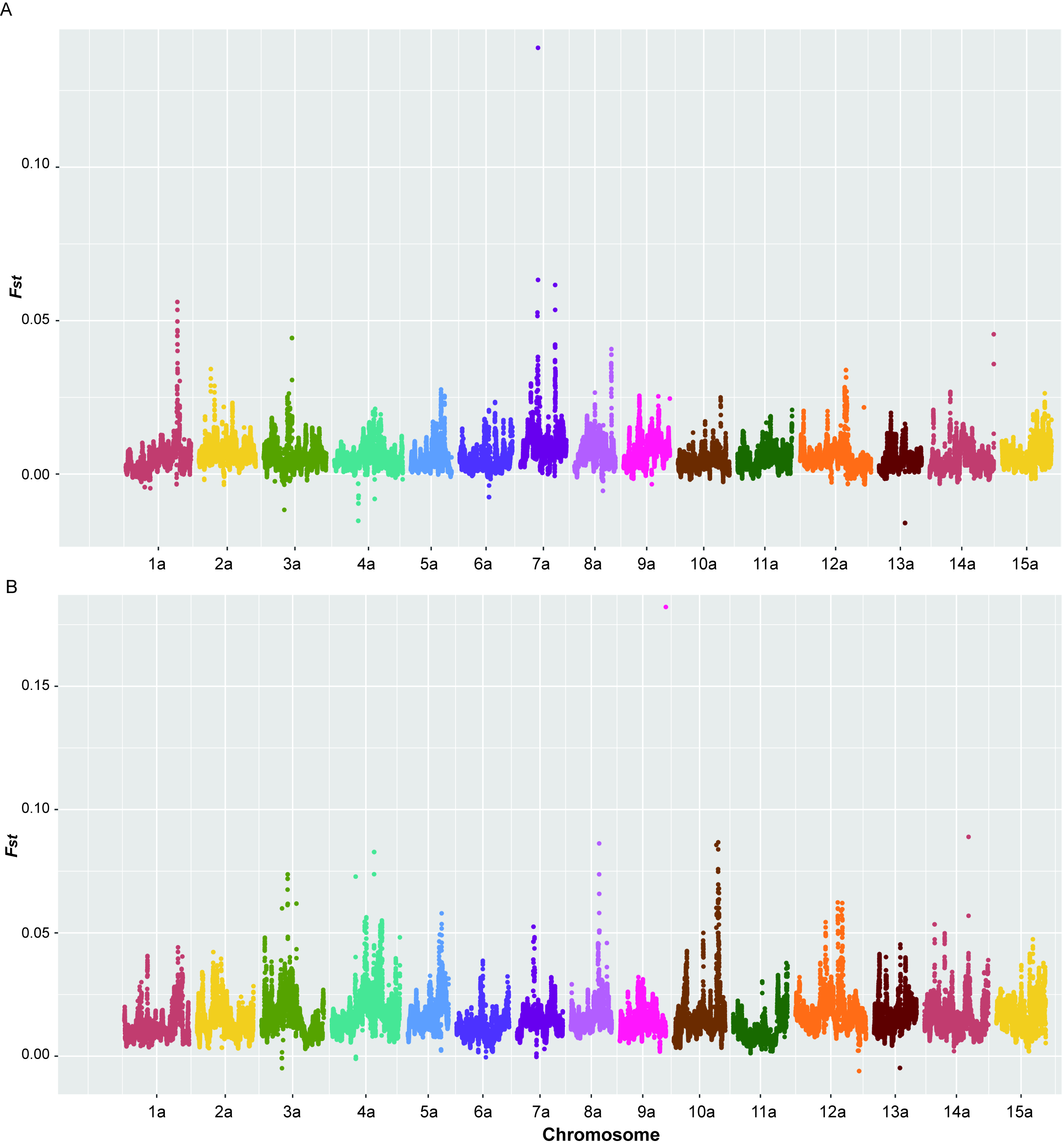

Supplement: Web_Material_uhac234 [file web_material_uhac234.zip › Fig.S3.tif]

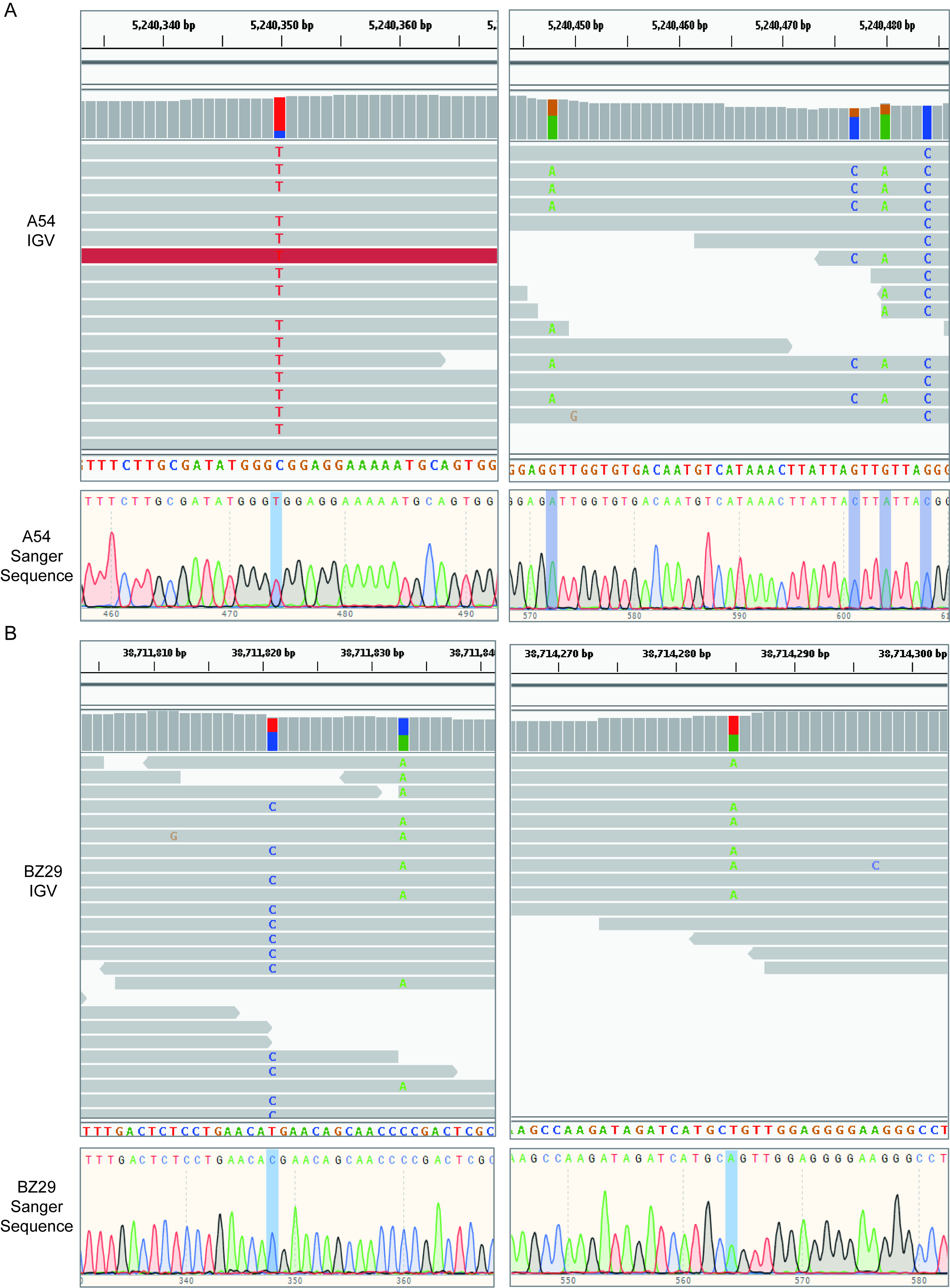

Supplement: Web_Material_uhac234 [file web_material_uhac234.zip › Fig.S30.tif]

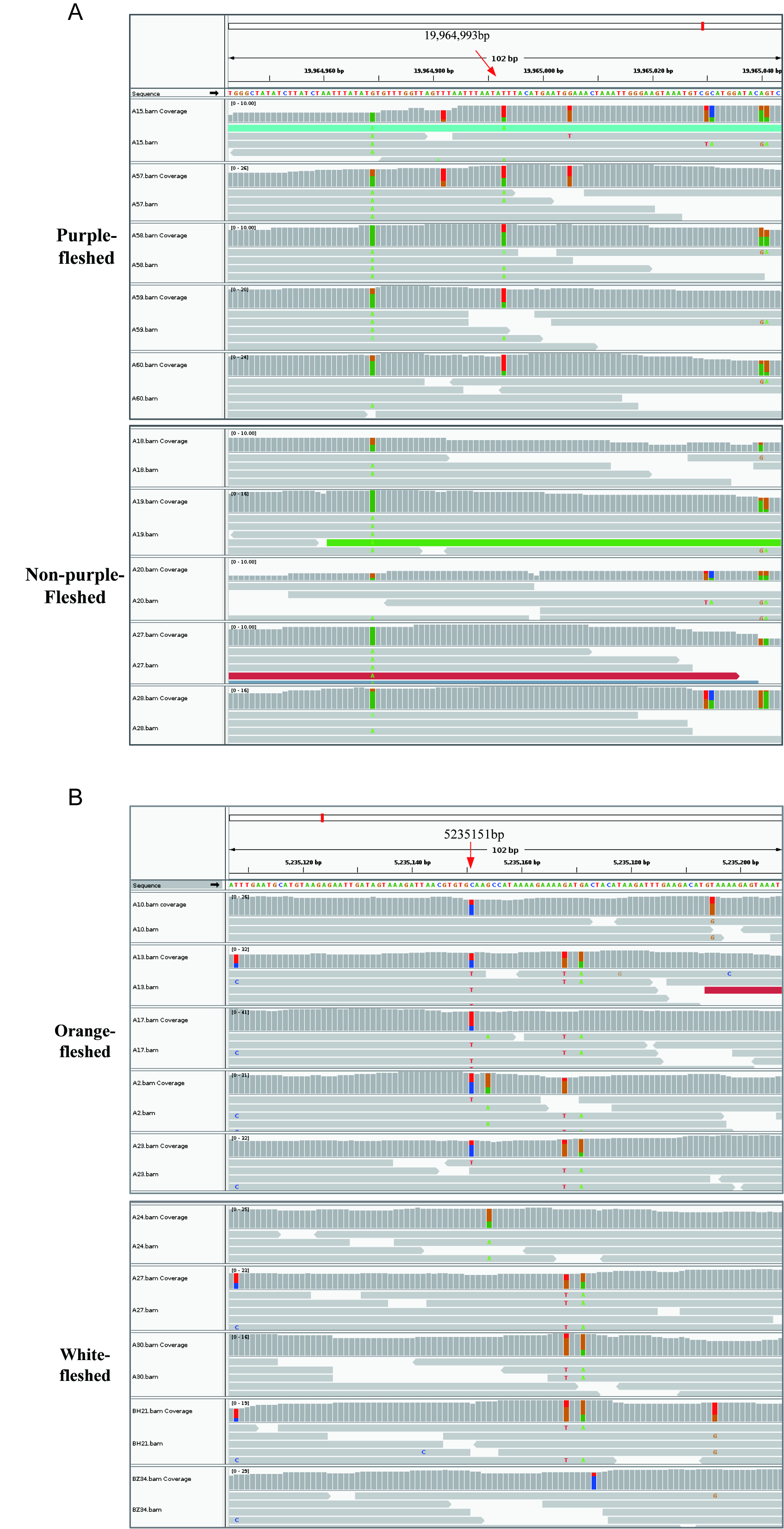

Supplement: Web_Material_uhac234 [file web_material_uhac234.zip › Fig.S31.tif]

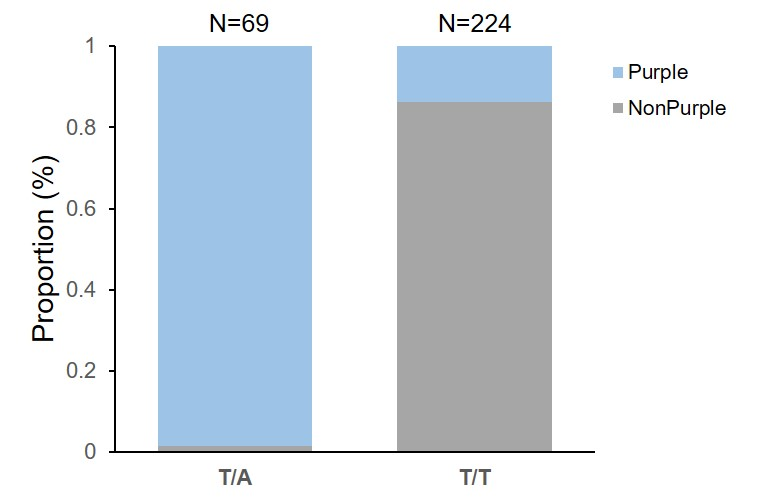

Supplement: Web_Material_uhac234 [file web_material_uhac234.zip › Fig.S32.tif]

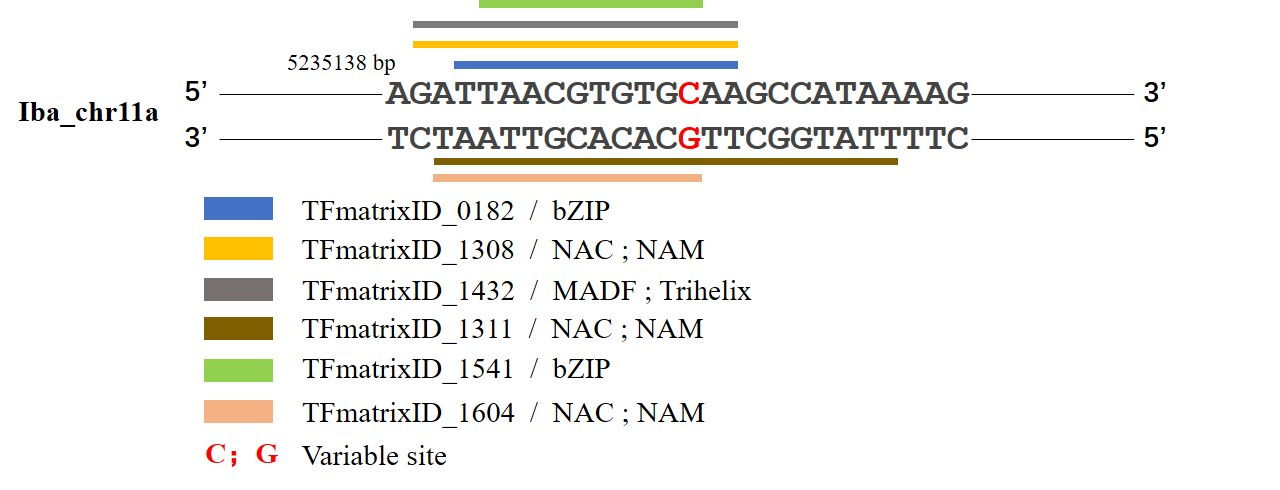

Supplement: Web_Material_uhac234 [file web_material_uhac234.zip › Fig.S33.tif]

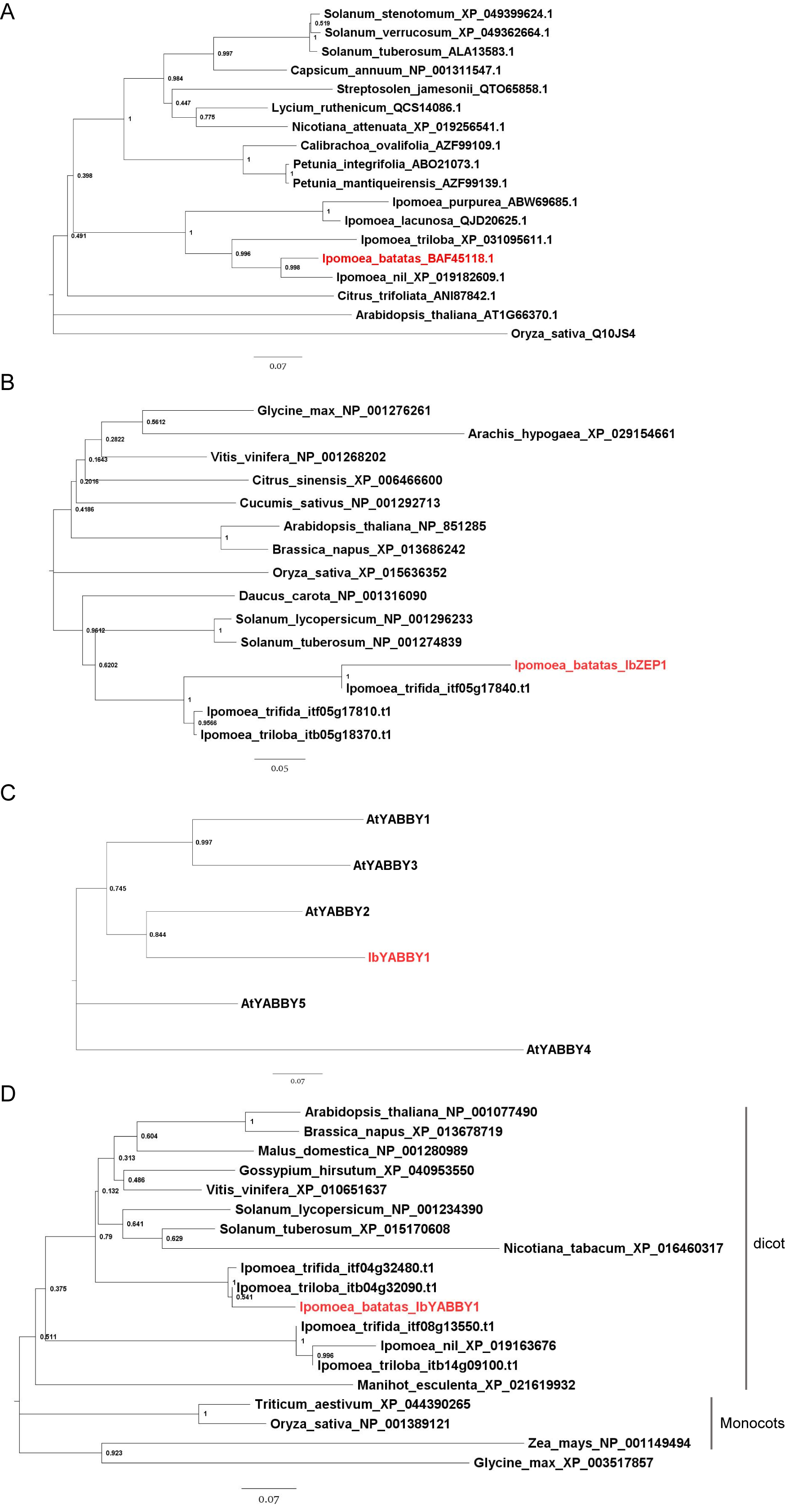

Supplement: Web_Material_uhac234 [file web_material_uhac234.zip › Fig.S34.tif]

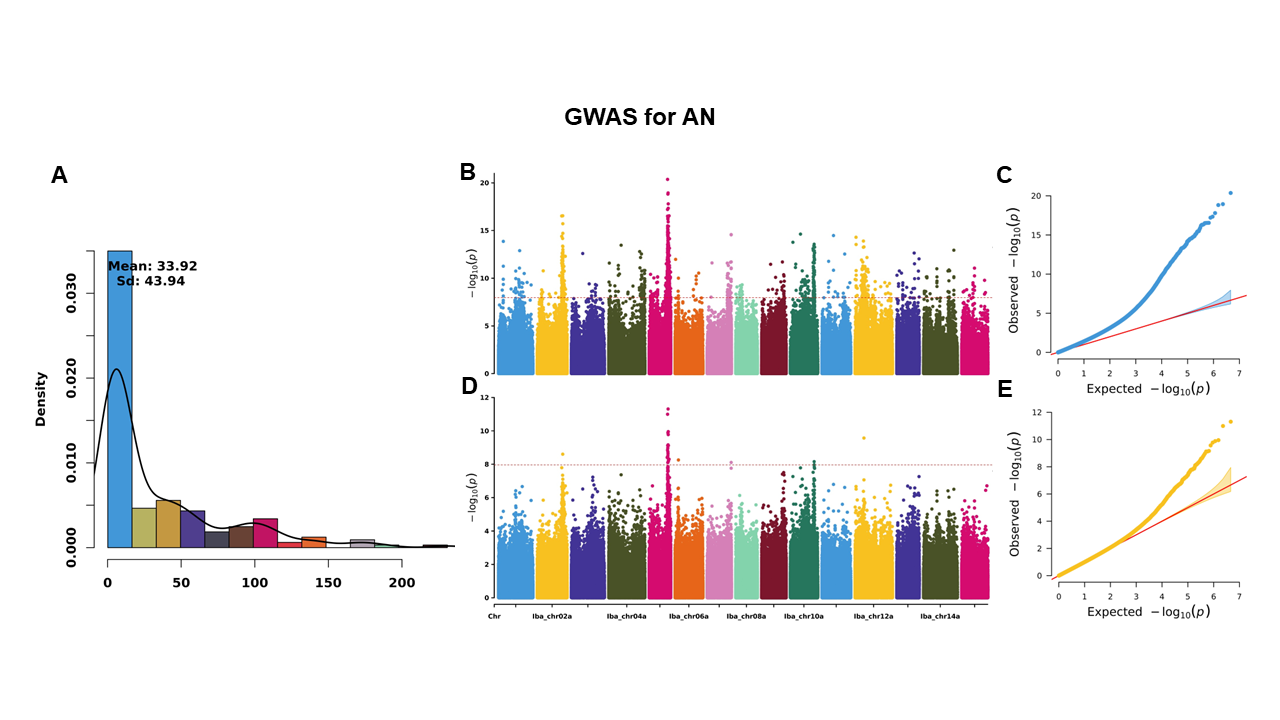

Supplement: Web_Material_uhac234 [file web_material_uhac234.zip › Fig.S4.TIF]

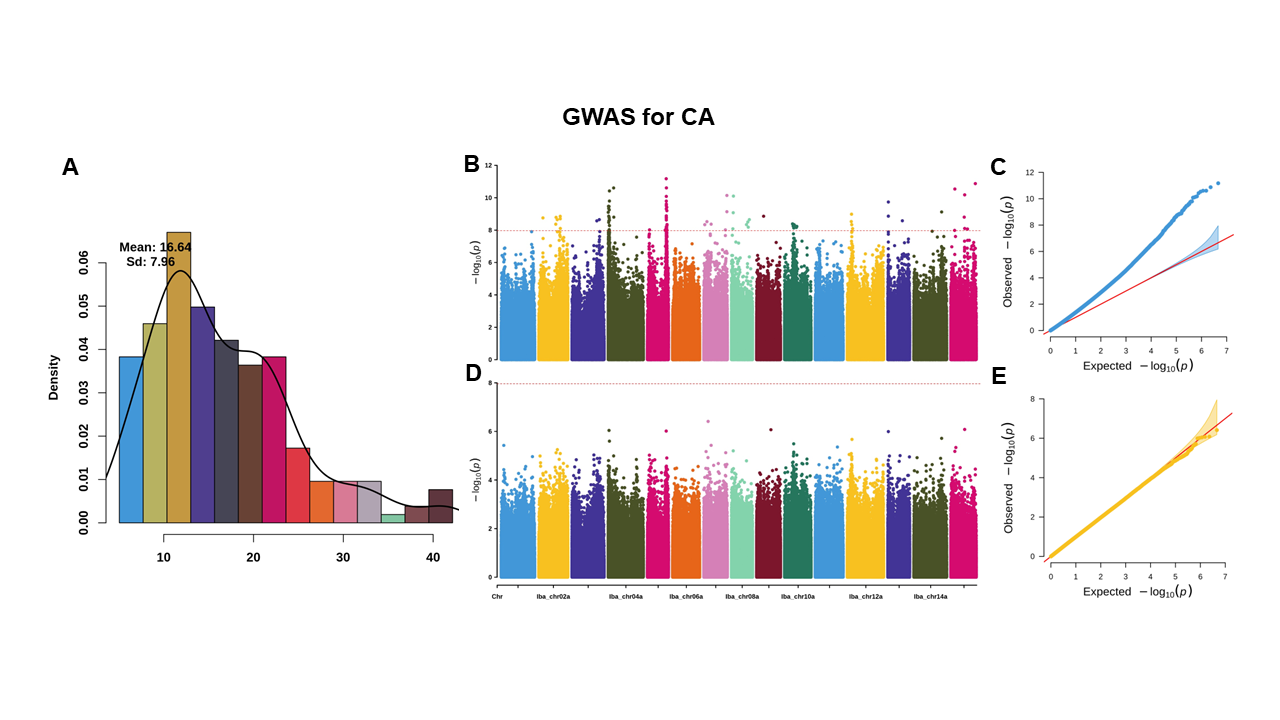

Supplement: Web_Material_uhac234 [file web_material_uhac234.zip › Fig.S5.TIF]

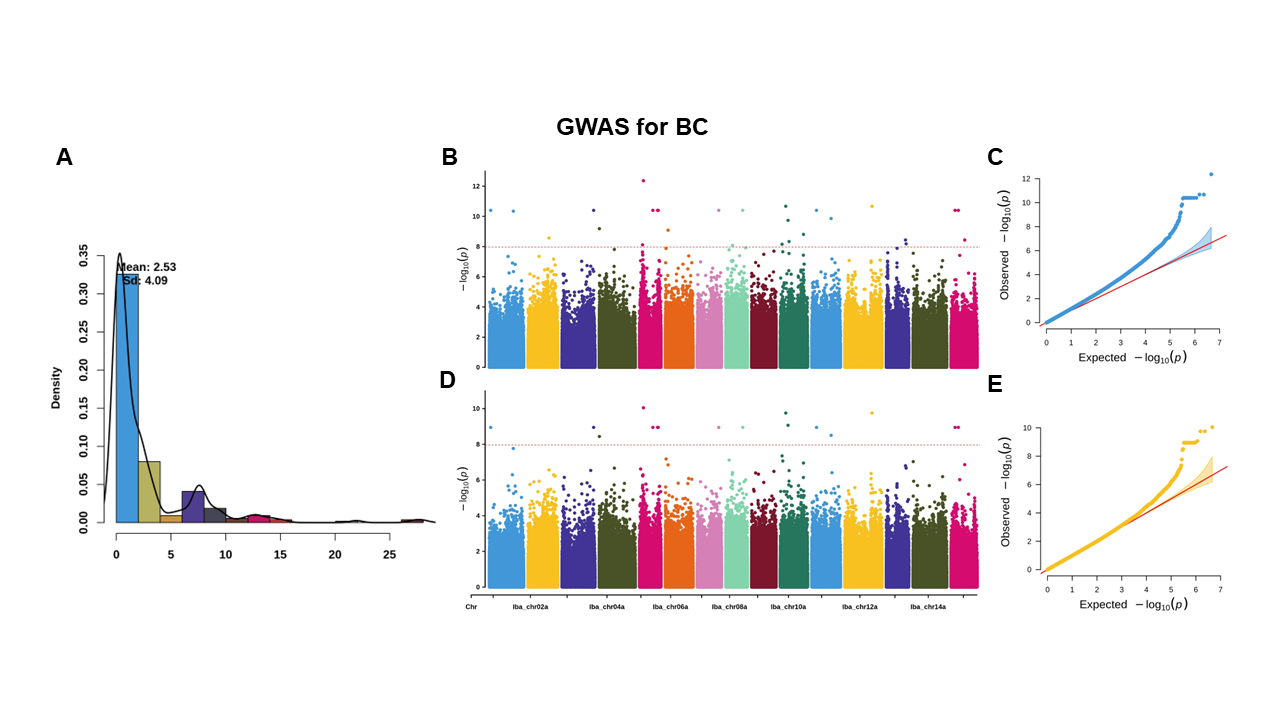

Supplement: Web_Material_uhac234 [file web_material_uhac234.zip › Fig.S6.TIF]

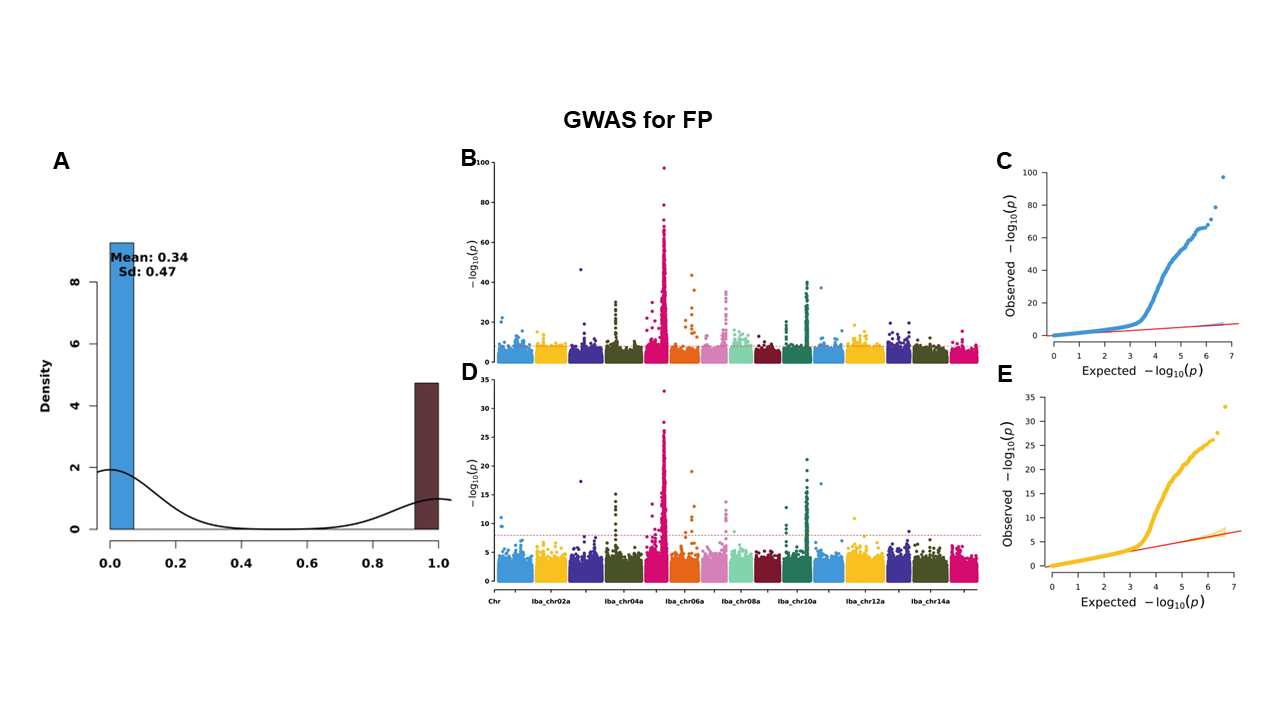

Supplement: Web_Material_uhac234 [file web_material_uhac234.zip › Fig.S7.TIF]

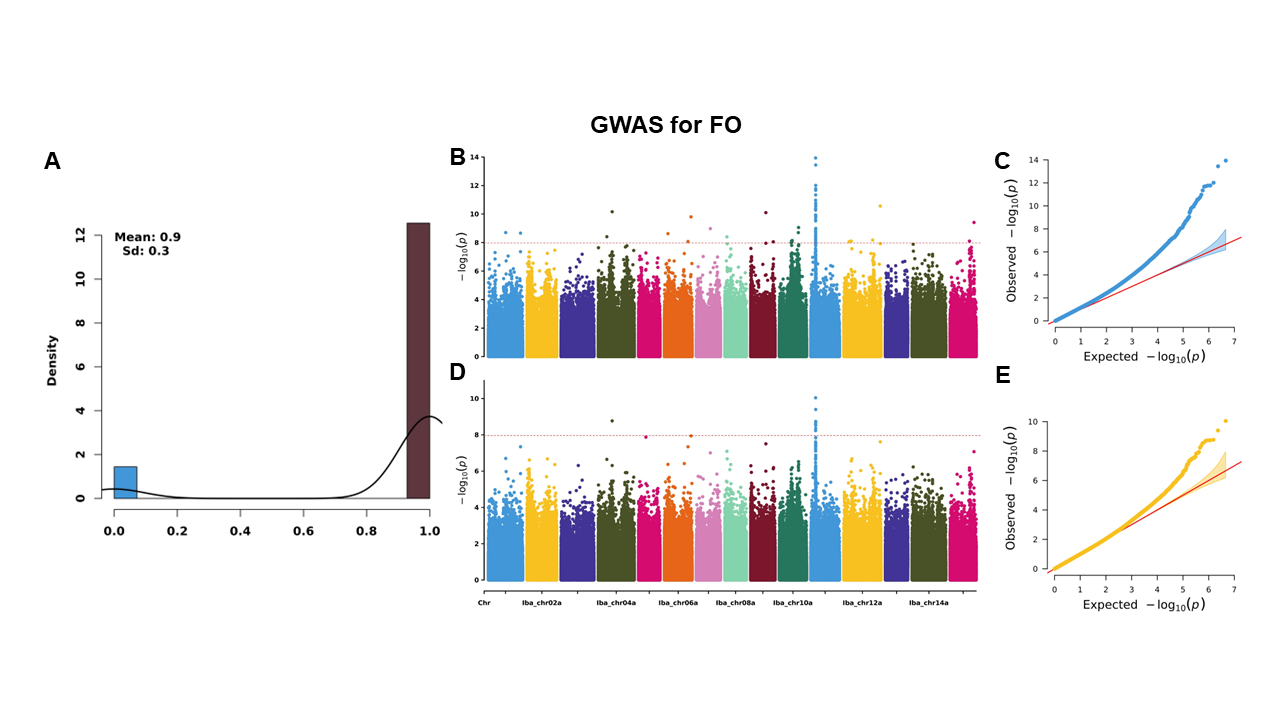

Supplement: Web_Material_uhac234 [file web_material_uhac234.zip › Fig.S8.TIF]

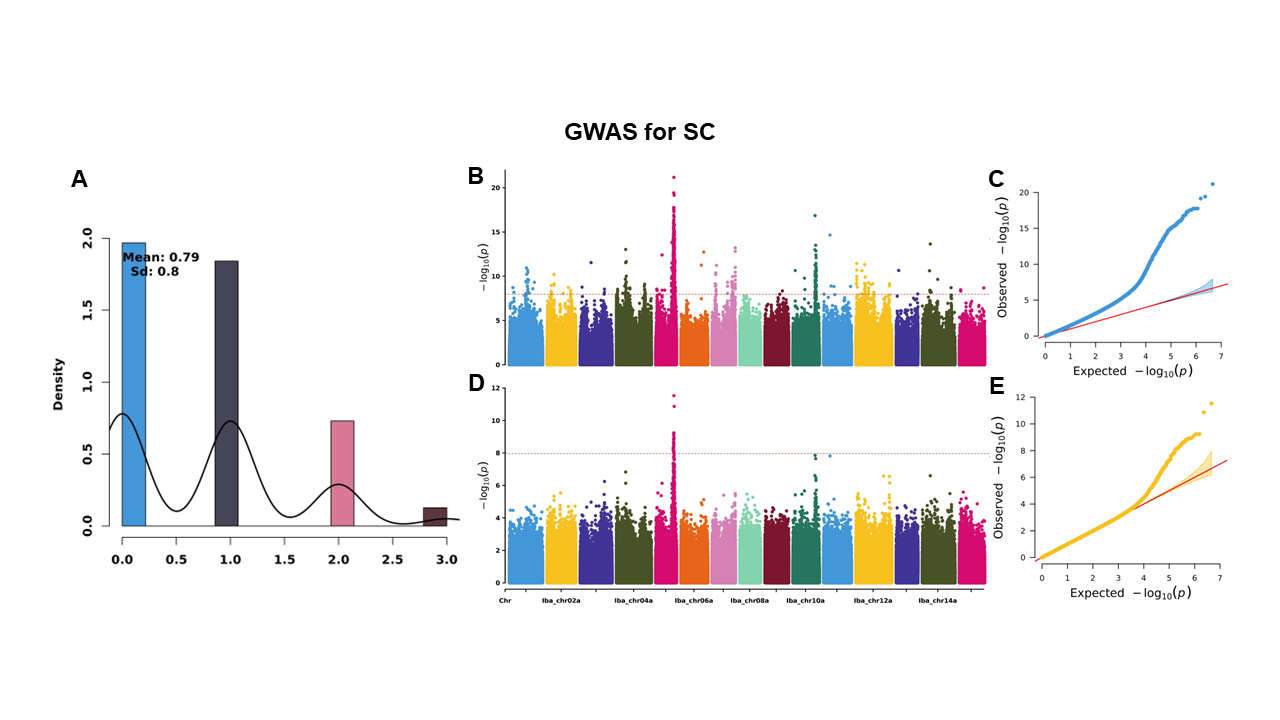

Supplement: Web_Material_uhac234 [file web_material_uhac234.zip › Fig.S9.TIF]
